# Tannin Gels and Their Carbon Derivatives: A Review

**DOI:** 10.3390/biom9100587

**Published:** 2019-10-08

**Authors:** Flavia Lega Braghiroli, Gisele Amaral-Labat, Alan Fernando Ney Boss, Clément Lacoste, Antonio Pizzi

**Affiliations:** 1 Centre Technologique des Résidus Industriels (CTRI, Technology Center for Industrial Waste), Cégep de l’Abitibi-Témiscamingue (College of Abitibi-Témiscamingue), 425 Boul. du Collège, Rouyn-Noranda, QC J9X 5E5, Canada; 2 Department of Metallurgical and Materials Engineering PMT-USP, University of São Paulo, Avenida Mello Moraes, 2463, Cidade Universitária, São Paulo CEP 05508-030, Brazil; gisele.amaral@usp.br (G.A.-L.); alan.boss@usp.br (A.F.N.B.); 3 Centre des Matériaux des Mines d’Alès (C2MA), IMT Mines d’Alès, Université de Montpellier, 6 Avenue de Clavières, 30319 Alès CEDEX, France; clement.lacoste@mines-ales.fr; 4 LERMAB-ENSTIB, University of Lorraine, 27 rue du Merle Blanc, BP 1041, 88051 Epinal, France; antonio.pizzi@univ-lorraine.fr

**Keywords:** tannin, polyphenolic molecules, sol-gel, organic gel, carbon gel, hydrothermal carbonization, porous materials, pore structure, biopolymer, low-cost

## Abstract

Tannins are one of the most natural, non-toxic, and highly reactive aromatic biomolecules classified as polyphenols. The reactive phenolic compounds present in their chemical structure can be an alternative precursor for the preparation of several polymeric materials for applications in distinct industries: adhesives and coatings, leather tanning, wood protection, wine manufacture, animal feed industries, and recently also in the production of new porous materials (i.e., foams and gels). Among these new polymeric materials synthesized with tannins, organic and carbon gels have shown remarkable textural and physicochemical properties. Thus, this review presents and discusses the available studies on organic and carbon gels produced from tannin feedstock and how their properties are related to the different operating conditions, hence causing their cross-linking reaction mechanisms. Moreover, the steps during tannin gels preparation, such as the gelation and curing processes (under normal or hydrothermal conditions), solvent extraction, and gel drying approaches (i.e., supercritical, subcritical, and freeze-drying) as well as the methods available for their carbonization (i.e., pyrolysis and activation) are presented and discussed. Findings from organic and carbon tannin gels features demonstrate that their physicochemical and textural properties can vary greatly depending on the synthesis parameters, drying conditions, and carbonization methods. Research is still ongoing on the improvement of tannin gels synthesis and properties, but the review evaluates the application of these highly porous materials in multidisciplinary areas of science and engineering, including thermal insulation, contaminant sorption in drinking water and wastewater, and electrochemistry. Finally, the substitution of phenolic materials (i.e., phenol and resorcinol) by tannin in the production of gels could be beneficial to both the bioeconomy and the environment due to its low-cost, bio-based, non-toxic, and non-carcinogenic characteristics.

## 1. The Chemistry of Tannins 

### 1.1. Definition and Classification

The utilization of tannins by human beings dates back to the second millennium before Christ, with leather tanning. The term ‘tannins’ itself is etymologically derived from the ancient Keltic lexical root ‘tan’, meaning ‘oak’ in reference to leather processing. After centuries of utilization, the chemical structures of tannins, their role, and their chemistry were extensively described for nearly half of century. Tannins are the most abundant compounds from the biomass after cellulose, hemicellulose, and lignin [1]. Their chemical structure is comprised of complex and heterogeneous polyphenolic secondary metabolites, biosynthesized by higher plants, with molar weights ranging from 300 g/mol for simple phenolic compounds to over 3000 g/mol for highly polymerized structures. Tannins are present in each cytoplasm of vegetable cells, and therefore in almost every part of plants such as barks, woods, leaves, fruits, roots, and seeds [2]. 

However, their quantity and composition may vary according to their vegetal source such as their botanic species, age, plant tissue, and environment. According to the species, a higher concentration has been reported in the wood of quebracho (*Schinopsis balansae* and *lorentzii*) and chesnut (*Castanea* sp.), in the bark of oak (*Quercus* sp.), pine (*Pinus* sp.) and mimosa (*Acacia mearnsii* formerly *mollissima* de Wildt), or in other tissues like in fruit pods of tara (*Caesalpinia spinosa*). Process extraction parameters (solvent, additives, temperature, time) are also a key factor in the composition of tannins extracts. Thus, the heterogeneous nature of tannins makes it impossible to settle on a universal method for their extraction, and imposes a reliance on relies on its final use for obtaining extracts [3]. According to their monomer unit, two wide classes of tannins exist: (i) hydrolysable tannins such as gallotannins (gallic acid compounds and glucose) and ellagitannins (composed of biaryl units and glucose), and (ii) condensed polyflavonoid tannins, this latter being stable and rarely subject to hydrolysis [4]. Some species produce exclusively either gallotannins, ellagitannins, or polyflavonoids whilst others produce a mix of all types of tannins. 

In their natural state, hydrolysable tannins are a mixture of simple phenols with a low level of phenol substitution and low nucleophilicity. The total world production of commercial tannins is estimated at 220,000 tons per year, with a large percentage of condensed tannins (>90%) available on the market [5]. With high reactivity and a relatively low price, condensed tannins are both chemically and economically more interesting for the preparation of adhesives, resins, and gels.

### 1.2. Condensed Tannins

**Figure 1:**
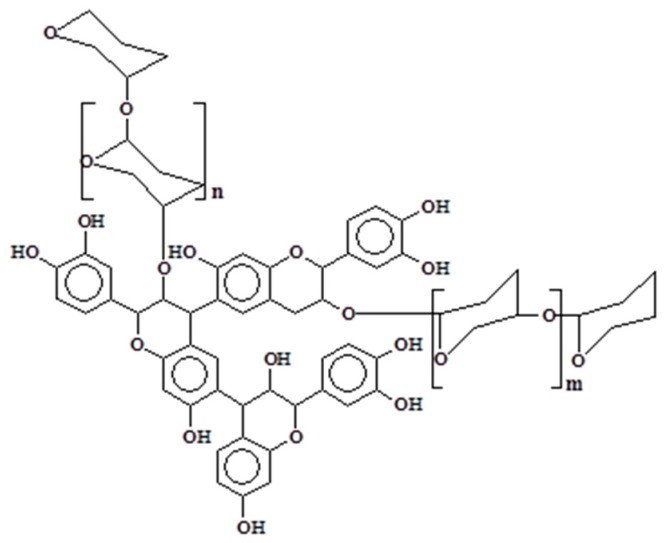
Polyflavonoids and carbohydrates linkage.

Condensed tannins are biosynthesized by the plant through their intermediate precursors (flavan-3-ols and flavan-3,4-diols) and other flavonoid analogs [6,7]. Their chemical structure is composed of flavonoids units that are subjected to various degrees of condensation. Traces of monoflavonoids or amino- and imino-acids are also reported in the composition of condensed tannins, but at too low concentrations to influence their chemical or physical properties [3]. However, other components in significant concentrations are often detected in tannin extracts which can modify the viscosity of the solutions. Among them, simple carbohydrates (hexoses, pentoses, and disaccharides) or carbohydrates chains of various length [8,9] can be linked to the flavonoid unit (Figure 1). Oligomers derived from hemicelluloses, complex glucuronates, and a low percentage of monoflavonoids (flavan-3,4-diols, flavan-3-ols, dihydroflavonoids, flavanones, chalcones, and coumaran-3-ols) could also be present in the extracts [6,7,10,11]. 

**Figure 2:**
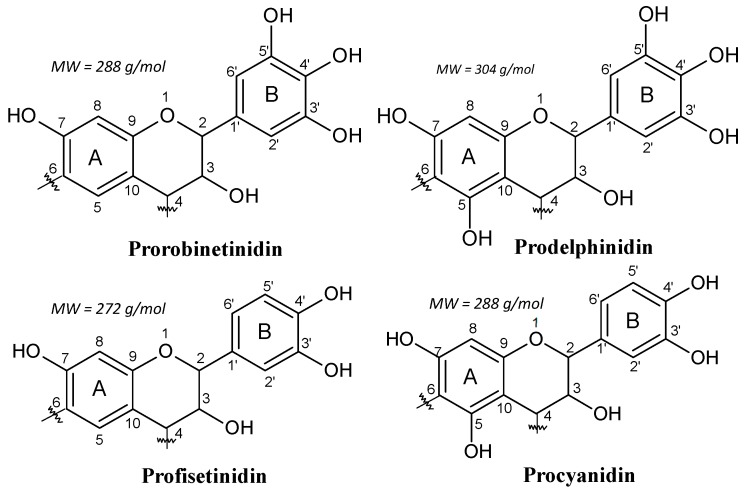
The four repeating flavonoid units in condensed tannins.

For example, in the black mimosa bark extract (*Acacia mearnsii*, formerly *mollissima*, de Wildt), it was reported 3−5% of monoflavonoids (flavan-3,4-diols and certain flavan-3-ols (catechin)) in its composition [7]. Thus, in this kind of tannin extract, each of the four combinations of resorcinol and phloroglucinol (A-rings) with catechol and pyrogallol (B-rings) coexist (Figure 2). In addition, the main polyphenolic pattern is represented by flavonoid analogs based on robinetinidin, and thus based on the resorcinol A-ring and pyrogallol B-ring. This pattern is reproduced in approximately 70% of the phenolic part of the tannin. The secondary but parallel pattern is based on fisetinidin, and thus on resorcinol A-rings and catechol B-rings. This represents about 25% of the total polyphenolic bark fraction. Superimposed on this two predominant patterns are two minor groups of A- and B-rings combinations.

These are based on phloroglucinol (A-ring)-pyrogallol (B-ring) (gallocatechin/delphinidin) and on phloroglucinol (A-ring)-catechol (B-ring) flavonoids (catechin/epicatechin). These four patterns constitute 65%−84% of commercial mimosa bark extract. The remaining parts of mimosa bark extract are the so-called “non-tannins”. This definition comes from the leather industry, where a “tannin” is considered to be any polyphenolic oligomer higher than, and comprising of, a trimer. It must be pointed out that the percentage of non-tannins varies considerably according to tannin extraction. The non-phenolic non-tannins can be subdivided into carbohydrates, hydrocolloid gums, and some amino and imino acid fractions [4,12].

### 1.3. Reactions of Condensed Flavonoid Tannins

**Figure 3:**
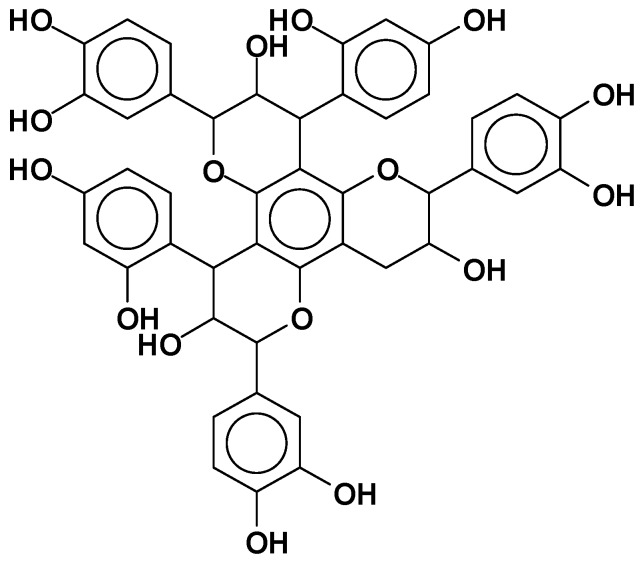
Chemical structure of phlobaphenes.

**Figure 4:**
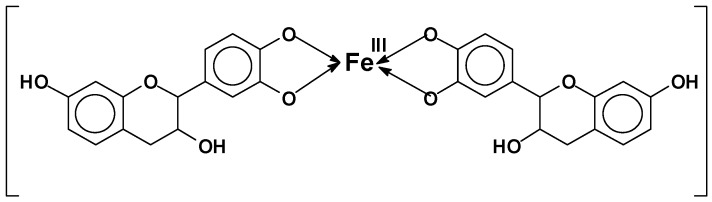
Ferric tannates [31].

Condensed flavonoid tannins are subjected to a number of reactions that impinge on their adaptability to different uses. These basic reactions in tannin chemistry have been abundantly described in the relevant review literature and the reader is recommended towards these reviews for more complete information [4,13,14]. The basic reactions of tannin are their rearrangements by:

1. Hydrolysis and acid or alkaline condensation [4,10,13,15,16,17]: This reaction leads to insoluble and unreactive compounds called “phlobaphenes” (Figure 3) or “tanners red” [18].
2. Sulfitation: This is one of the older reactions used in tannin chemistry to decrease the tannins viscosity in water and improve their water solubility [4,19], but the excess of sulfite can be deleterious for some applications [20].
3. Catechinic acid rearrangement: While this rearrangement is easily shown to occur in model compounds where the reaction is carried out in solution [13,21], it is much less evident and easily avoidable in tannin extracts where the colloidal nature of the extract markedly limits its occurrence. This is fortunate, as otherwise some fast-reacting tannins such as pine, pecan, cube gambier, etc. could not be used to produce resins, adhesives, and other thermosetting plastics [16,22,23,24,25,26].
4. Catalytic tannin self-condensation: Polyflavonoid tannins have been found to self-condense and harden when in the presence of particular compounds acting as catalysts. Foremost, there is the catalytic effect of small amounts (2%−3%) of silica smoke, or nanosilica or silicates at high pH [27]. This reaction is fast and markedly exothermic, where a concentrated solution of tannin at 40%−50% in water gels and hardens at pH 12 and 25 °C in 20–30 min. The strong exothermic character of the reaction leads to this result, as the temperature increases by several tens of degrees in a short time [27]. Small amounts of boric acid and AlCl_3_ were found to have the same effect but are much less exothermic.
5. Tannin complexation of metals: Tannins readily complex metal ions [28]. This characteristic is used to capture or precipitate toxic metals in water [29,30] and to isolate a rare metal such as germanium from its copper matrix, where it is mined for paint primers for metal application and several other applications. An old example is the formation of iron complexes, used to prepare intensely black/violet inks by the formation of ferric tannates (Figure 4). These coordination complexes are due to the ortho-diphenol hydroxyl groups on the tannin B-rings.
6. Reactions of tannins with formaldehyde and other aldehydes: Due to their phenolic nature, tannins undergo the same alkali- or acid-catalyzed reaction with formaldehyde as phenols (see more details in Section 2.2.1.).
7. Reactivity and orientation of electrophilic substitutions of flavonoids such as reaction with aldehydes: The relative accessibility and reactivity of flavonoid units is of interest for their use in resins, adhesives, and gels. A detailed description of reactions at different pHs is given in Section 2.2.2.

**Figure 5:**
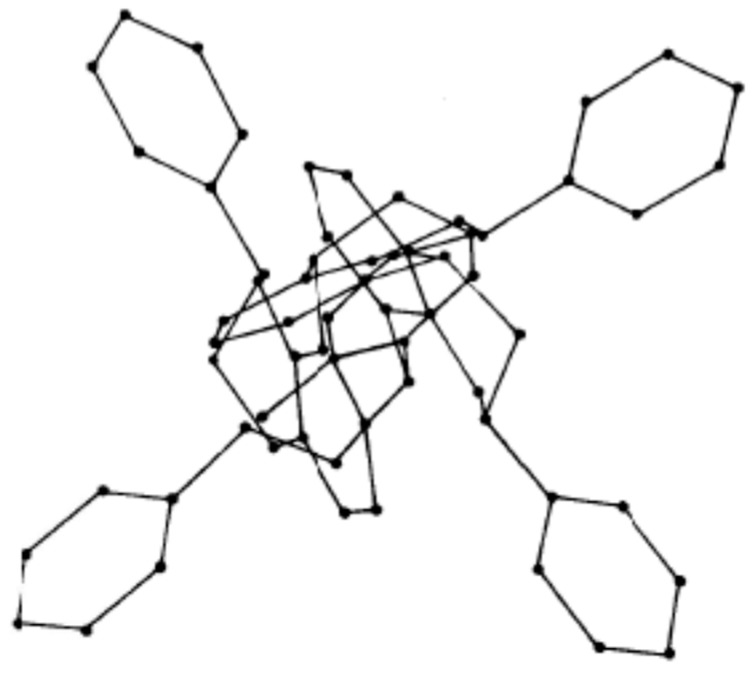
Tridimensional structure of a tetraflavonoid 4,8-linked.

While there is an abundant literature in chemical journals about tridimensional structure of flavonoid monomers, there is a scarcity of research in the literature about the three-dimensional spatial configuration of flavonoid oligomers. There is only one molecular mechanism study on this topic [32]. This study shows the correlation between the applicability of these materials and their 3D structure. For example, for a tetraflavonoid of 4,8-linked catechins, all 3,4-cis is in the helix configuration and when observing the helix axis, a characteristic structure presenting all four B-rings pointing outwards appears (Figure 5). Such a structure, rendering the hydroxyl groups of the B-rings particularly available, obviously facilitates their use and reactions, such as adhesion to a lignocellulosic substrate, formation of metallic coordination complexes [28,29,30], formation of polyurethanes with and without isocyanates [33,34], and other outcomes where the reaction of the B-ring is of interest [35], such as cross-linking at pH 10 and higher. 

Water solutions of 40%–50% polyflavonoid tannin extracts appear to be in a colloidal state as indicated by their zeta-potentials [36], this has been confirmed by ^13^C NMR. This is caused by both the presence of noticeable proportions of hydrocolloid gums (fragments of hemicelluloses) as well as the presence of higher molecular mass tannins. 

### 1.4. Industrial Extraction 

Condensed flavonoid tannins are generally obtained from crushed bark or wood chips through countercurrent industrial hot water extraction (70–90 °C), but not under any pressure. Small percentages of sodium sulfite or metabisulfite can be added, sometimes with even lower proportions of sodium bicarbonate to improve solubility of oligomers of high molecular weight tannin. The sulfitation of tannins during their extraction, or after extraction, is one of the oldest and most useful reactions in the chemistry of condensed tannins. This process may be useful, but harmful if done excessively, depending on the end use for which the tannin extract is intended. Sulfitation allows the preparation of tannins of lower viscosity and increased solubility, which are therefore easier to handle [4,13]. After having passed through stainless steel tanks, a fine aqueous solution of tannin at very low concentration is first obtained, then concentrated to about 35% solids and spray-dried, or concentrated under vacuum to 86% concentration and bagged to form a solid mass, the so-called tannin extract “cast” [18]. 

The type of extraction process defined above has been used since the beginning of the 20th century for extracts of mimosa (*Acacia mearnsii*) and quebracho (*Schinopsis* sp.), which are the world’s two largest sources of commercial condensed tannin extracts. The percentage yield of tannin extract is about 28–33% of the original weight of the bark or wood for these two species, which makes the extraction very profitable. Procyanidin or prodelphinidin tannins present generally higher molecular weight fractions and can undergo internal rearrangements, resulting in lower extraction yields of about 12−15%. Addition of a small percentage of urea to conventional solutions used for extraction has led to a higher yield level of 18–25%, and thus increased their economic interest [18]. Extraction by organic solvents, although giving higher yields, has proved to be expensive and unacceptable at the simple technological level of plant extraction. In addition, there are regularly percentages of other materials in the extracts, thus posing the problem of further purification of the tannin extract. In cases in which tannins are used for nutritional or pharmaceutical purposes, a second purification with organic solvents is necessary to remove the carbohydrates fraction present in the extract. 

### 1.5. Industrial Uses of Condensed Tannins

There are several established industrial uses of condensed tannins, some of long date, some more recent. It is too long to explain in detail here, and the reader is invited to read relevant reviews on different subjects [31,37]. Only a few lines will be spent here to enumerate them in a very brief description. Traditionally, tannins have been used for (i) Leather tanning, the oldest and still the most important industrial applications, where tannin extracts have been used since the last quarter of the 19th century. However, tannin-containing bark had been used for this application since the second millennium before Christ [4]; (ii) Wood thermosetting adhesives, possibly the second largest use of tannin, but still far behind leather. This use takes up in part the slack of the leather market since the 1970s, but with new generations of wood adhesives still appearing [6]. Added adhesives for corrugated cardboards and for foundry sands must be used for this process [38]; (iii) Pharmaceutical applications, these being traditionally centered on troubles for the digestive system; (iv) Depressors of calcite ore flotation [8]; (v) Flocculants for the precipitation of heavy metal and other pollutants in water [11]; (vi) Stabilizers for oil and other deep level drilling; (vii) Additives for wine, beer and fruit juices; (viii) Ferric tannate writing inks, one of the oldest uses started from the 15th century [11]; (ix) Superplastifiers for cement [13]; and (x) metal antirust primer varnishes, this industrial usage that was in vogue in the 1950s and 1960s is now experiencing a revival of interest [4].

In addition to this more traditional long time operational, or even reasonably more recent usage, there is an increasing interest in researching new materials and applications for condensed tannins. At present, the interests that are more highly researched and focused on are (i) Fire resistant foams for several applications, such as thermal and acoustic insulation [39,40,41], as well as for hydroponic cultivations [19,42,43,44]; (ii) Hard thermosetting plastics for angle grinder disks and car brake pads [45,46]; (iii) New medical applications such as antiviral drugs and support for stem cells for bone reconstruction [8,47]; (iv) Polyurethanes with and without isocyanates for surface finishes and other applications [33,34]; (v) Adhesives for Teflon coatings on aluminum and steel for high temperature resistance [11]; (vi) Epoxy resins [48,49]; (vii) Flexible films [50] and flexible gels [51]; (viii) Fiber panels impregnated of tannin resins [18]; and (ix) Tannin-based gels, which will be extensively discussed in the next session.

## 2. Tannin Gels

### 2.1. Tannin Gels Synthesis

**Figure 6:**
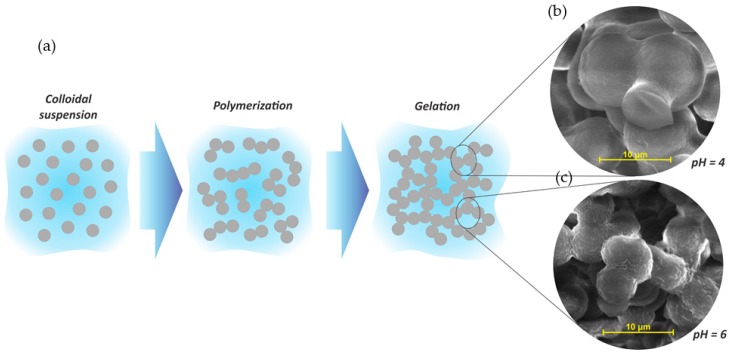
(**a**) Schematic representation of sol-gel process of tannin gels and their respective SEM images at pH 4 (**b**) and pH 6 (**c**). Adapted with permission from reference [61]. Copyright 2013 Elsevier.

Organic porous tannin gels are versatile materials that can be used for several applications, mainly because of their final characteristics, which are normally tailored during synthesis steps. The tannin gel synthesis is based on a sol-gel process of soluble precursors. The colloidal suspension of solid particles (sol) is composed of tannin and a crosslinker dispersed in a solvent, commonly water. A schematic representation of a sol-gel process is given in Figure 6a.

Usually, the reactions start with an addition reaction between the main precursor (tannin) and the crosslinker (aldehyde), making these species more reactive. The partially hydrolyzed particles then crosslink by polycondensation reactions, releasing water molecules. Thus, the polymeric chains grow, forming a giant cluster of macroscopic size, becoming insoluble. This phenomenon is known as gelation [52]. The sol-gel transition is usually estimated by a significant change in viscosity through visual observation of the container tubes where the solutions are placed. Normally, reactions happen in a sealed flask at moderate temperature (50–85 °C), so that the whole solution can be converted into a gel [52], and when the viscous liquid is no longer flowing at an angle of 45°, the solution is considered gelled [53,54], establishing the gelling time (T_gel_) [55]. The polymerization reaction in the aqueous medium generates a gel as a semi-solid system containing two phases: a solid based on a nanostructured network (spherical nodules) interconnected by narrow necks defined as "string-of-pearls", which is enclosed in a high porosity inter-penetrated solvent medium (second phase), as well as in some by-products formed during the polymerization reactions [52,56,57]. The size of these nodules depends directly on the synthesis conditions, especially the pH and the mass fraction of solids in the initial solution [58,59,60,61]. Usually, phenolic gels prepared under acid conditions lead to a tridimensional chain with large nodules, consequently developing larger pores and higher pore volumes [60,61,62,63]. By increasing the pH to an alkaline level, a less porous material is created, with smaller pores or non-porosity [60,61,64]. Since tannin gels are porous materials from a phenolic precursor, they also follow this trend, as presented in Figure 6b,c. 

After gelation, the polymerization reactions still continue, since the network formed is highly flexible and its constituent chains can move in relation to each other [62]. At this point, small clusters still exist and covalent bonds cross-linking occurs within the main network [52,55,62]. Besides, a fraction of the water in the polymerized gel structure is present as methylol derivatives as −OH groups [62], and part of the porosity is still prone to evolve, since evaporation of water generates voids within the network. Therefore, gel drying should not be done right after gelling because an ageing step is required to ensure the maximum formation of crosslinks [52,62,65]. These additional reactions generate a porous material with better mechanical properties, i.e., are able to withstand capillary forces during the drying process [62].

The tannin gels formed are classified as physical or chemical, according to the type of crosslinks in their network structure [60]. The so-called physical gels are formed by weak bonds, usually based on Van der Waals and other secondary forces [66] such as hydrogen bonds [67]. Chemical gels instead have a structure based on strong covalent bonds crosslinks, establishing a reticulated network and an infused gel [67]. 

### 2.2. Mechanisms

#### 2.2.1. Tannin-Formaldehyde-Systems 

**Figure 7:**
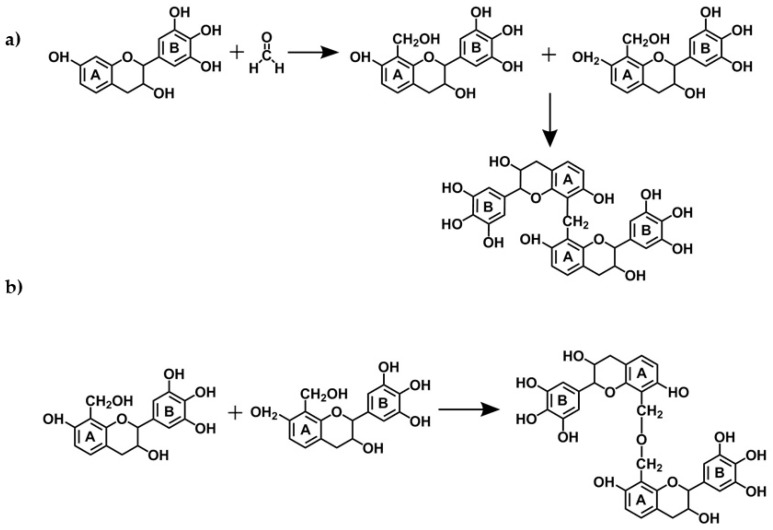
Reaction mechanisms of a condensed tannin monomer with formaldehyde resulting in: (**a**) methylene bridges and (**b**) methyne ether bridges.

The reactions of tannin gels are based on polymerization of flavonoids units from condensed tannins with formaldehyde (the most used aldehyde for the production of tannin gels), especially with the flavonoids A-rings through methylene bridge linkages [14,68,69]. However, experimental studies suggest that the B-rings (catechol or pyrogallol) are also able to react with formaldehyde in a more reactive medium, with more acidic or alkaline [70], or with the addition of zinc acetate [71,72,73,74,75]. The main cross-linking reactions involving tannin-formaldehyde systems are the formation of both methylene bridges (−CH_2_−) and unstable methylene ether bridges (−CH_2_OCH_2_−) as shown in Figure 7. The latter is unstable and thus easily rearranged, forming methylene bridges and releasing formaldehyde [14]. Additionally, carbohydrates and complex glucuronates present in tannin extracts also react with formaldehyde, which might assist in network formation [14]. During the formation of the tannin-formaldehyde network, the initial immobilization of the network places far from each other a number of potentially reactive sites thus preventing the formation of further methylene bridges. Substitution of formaldehyde by other aldehydes, by up to 30%, improves the resin cure. The reactions of tannins with aldehydes that have led to their extensive applications as wood adhesives will not be discussed here, since extensive reviews can be found elsewhere [14,42,70].

#### 2.2.2. Influence of pH and Mass Fraction

**Figure 8:**
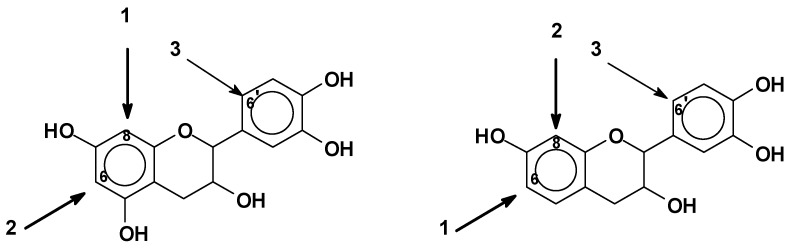
Reactive sites of flavonoid units.

As mentioned before, pH control is extremely important in the reactional system of phenolics. The C8 site on the A-ring (Figure 2) is the first one to react, e.g., with an aldehyde, and when free, it is the site with higher reactivity [10,70]. The C6 site on the A-ring is also very reactive, but less than the C8 site, since this latter presents lower steric hindrance [10,70]. Generally, the reactions involve only these two sites on the A-ring. The B-ring is particularly unreactive. A low degree of substitution at the 6’ site of the B-ring can occur (Figure 8). In general, at higher pHs such as pH 10, the B-ring starts to react too, contributing to cross-linking as well [68,76]. Thus, for catechins and phlorogucinol A-ring type flavonoids, the reactivity sequence of sites is C8 > C6 > C6’ when these are free. For robinetinidin and fisetinidin, thus for resorcinol A-ring type flavonoids, the reactivity sequence is modified to C6 > C8 > C6’ due to the greater accessibility and lower possibility of steric hindrance of the C6 site (Figure 8) [10,68].

The T_gel_ is a useful parameter to confirm the precursor reactivity at different pHs. The curve of T_gel_ of flavonoid tannins with aldehydes has always the shape of a bell curve. Normally, the longest T_gel_ is around pH 4, while the fastest ones are at lower and higher pHs, depending on the tannin system studied. The curve reaches an almost asymptotic plateau of very high reactivity and short T_gel_ around pH ≥ 10 and at pHs < 1−2 [42,70]. The maximum T_gel_, i.e., the reaction at the lowest condensation rate, changes as a function of both the temperature and the reaction system. Thus, tannin-formaldehyde gel has the highest T_gel_ at pH 4−5 (50 °C) [77].

During their polymerization reactions, the tannin molecules may become inaccessible to reagents due to the network early immobilization and tridimensional structure, as mentioned before. Such a steric hindrance is associated with a lack of flexibility, especially when the network tannin-formaldehyde forms already at low degree of condensation. In this case, the prospective residual reactive sites become too distant to participate to the cross-linking leading to incomplete polymerization [13].

Mass fractions of total solids from initial solutions (Equation (1)) also represent an important parameter in the production of tannin gels. In the system tannin-formaldehyde, for example, low concentrations of mass fraction (˂18 wt.%) induce gels with high pore volumes. However, the low final density results in a fragile structure, subjected to a higher shrinkage during the drying process [60] and partial porosity losses. Increasing the mass fraction of solids from the initial solution (22–40 wt.%) tends to produce gels more consistent, but with less porosity [60]. It is important to notice that there is always an optimal condition depending on the final textural properties required. Thus, the experimental parameters as pH and mass fraction should be optimized and chosen to fit the desired application.

(1) $$Mass\;fraction\;\;(\%)=\frac{m_{solids}}{m_{solids}+m_{solvent}}$$

#### 2.2.3. Tannin-Soy-Formaldehyde Gels

**Figure 9:**
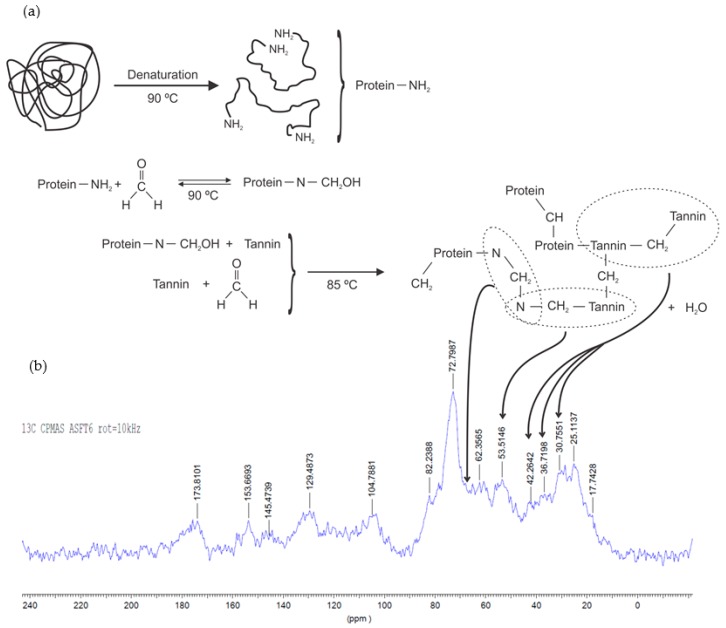
Suggested cross-linking reactions on tannin-soy-formaldehyde gel (**a**) and ^13^C-NMR spectra of organic gel at pH 6 (**b**) [54].

Organic gels can be produced using only tannin as the raw biopolymer, or this can be combined with a second biosourced material, such as lignin [78] or protein from soy flour [54]. Soy-tannin-formaldehyde is known as an adhesive for wood particleboard [79]. Other natural materials such as albumin have been already applied for the production of a macroporous monolith (tannin-albumin-formaldehyde) [80]. At the same time, diluted adhesives might be explored in the preparation of gels [81,82]. A reaction mechanism was proposed for tannin-soy-formaldehyde gels (Figure 9a) [54]. The authors showed the main reactions occurring using FTIR, ^13^C-NMR, and XPS analyses. First, the soy protein is denatured to expose its amide groups (N), followed by an addition reaction with formaldehyde, and finally by a copolymerization with the tannin (T). The main cross-linking reactions occur between tannin-soy (N‒CH_2_‒T) or soy-soy (N‒CH_2_‒N) and tannin-tannin (T‒CH_2_‒T) through methylene bridge linkages, as demonstrated by ^13^C-NMR spectra (Figure 9b). The reactivity of tannin-soy-formaldehyde gels slightly changes, so its highest T_gel_ was found to be at pH 6 and 85 °C [54] compared to pH 4–5 and 50 °C for tannin-resorcinol-formaldehyde gels [53]. 

### 2.3. Preparation Methods and Conditions

#### 2.3.1. Hydrogels Formulations at Normal Conditions 

After dissolution of all reactants, the final solution is usually placed in a hermetically sealed container to avoid evaporation of liquid, and then kept at determined temperatures (50–85 °C) over five days for gelation and ageing. The latter conditions used during the preparation of tannin gels will be considered in this review as the “normal conditions”. After this period, materials were left to cool down at room temperature before the solvent exchange step. It is worth noting that materials prepared with no ageing or temperature steps are also referred as tannin gels in the literature. Normally, the authors synthesized gels by mixing tannin with formaldehyde, leaving them for several hours at room temperature, and drying them at temperatures higher than 45 °C to generate a final product with a gummy aspect [83,84,85,86,87,88]. However, those gels are much less porous (e.g., up to 6 m^2^/g [88]) but largely employed as adsorbents for organic and inorganic contaminants in water treatment at a laboratory scale (see Section 2.6). Thus, these materials will be termed as *tannin-gel* in this review to differentiate the highly porous gels prepared after the gelation, ageing, and drying steps.

The majority of tannin gels prepared in presence of an aldehyde at a different mass fraction, mass ratio, and pH are considered as tannin chemical gels. However, physical tannin-based gels can also be obtained by varying the mass fraction and the pH in the following proportions, e.g., 4 wt.% at pH 6 and 10 wt.% at pH 8. In such conditions, the solutions are gelled only after been removed from the oven (after cooling down) but they become a liquid again when they are returned to the oven (melting point around 85 °C). This phenomenon is not been fully understood [60]. 

#### 2.3.2. Solvent Exchange

The tannin-based gel so prepared is named after the solvent employed during its synthesis. Hydrogel or aquagel fits for water, while alcogel is applied for gels prepared in alcohol medium [89]. The formed solid is an organic macromolecule, which has saturated pores with (i) Water and/or solvent; (ii) Unreacted residual products; and possibly (iii) By-products of the polycondensation reactions. The liquid in the pores must be replaced by air through a non-destructive procedure, ensuring that the formed nanostructure is not destroyed [90] and most of the porosity is preserved [91]. 

To do so, an intermediate procedure is required. The produced hydrogel is subjected to a solvent exchange, i.e., the liquid present in the porosity (water, alcohol, by-products) is replaced by an appropriate solvent accordingly to the desired drying method. Solvents such as acetone [77], ethanol/carbon dioxide [60], or *tert*-butanol [53] are usually employed. In the literature, most authors exchange their hydrogels for three days, replacing the solvent every day with a fresh one and providing an effective solvent exchange within the tridimensional structure of the gel [60,77].

#### 2.3.3. Drying Conditions and Final Physicochemical Properties of Tannin Gels

After solvent exchange, tannin gels are finally ready to be dried. There are three types of drying procedures that are commonly used in the production of gels, which result in different porous materials with distinct textural properties:

1. Subcritical drying: Gels are dried under atmospheric conditions to form xerogels.
2. Freeze-drying: Gels are dried at freezing conditions to produce cryogels.
3. Supercritical drying: Gels are dried at a critical point of a working fluid to produce aerogels.

**Figure 10:**
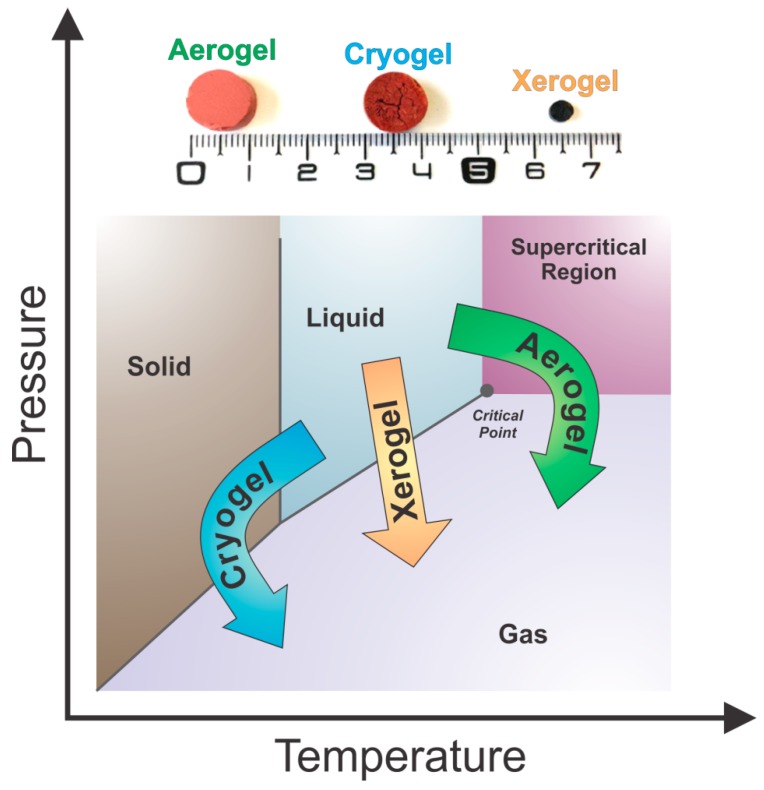
Phase diagram of the solvent within the gel structure and the representation of the different drying methods with their respective porous materials, adapted with permission from reference [92].

The respective dried materials from a gel prepared from tannin and formaldehyde with a resin mass fraction of 6 wt.% and an initial pH of 2 (aerogel, cryogel and xerogel) are presented in Figure 10 [92]. Visually, it is possible to notice macro differences: (i) Xerogel presents a considerable shrinkage; (ii) Cryogel shows micro cracks due to the formation of ice crystals coming from solvent during the freezing stage; and (iii) Aerogel presents a better preservation of the initial volume and porosity. More details about each of these methods are described below.

Subcritical drying allows the evaporation of solvent at room temperature, or using an oven at temperatures up to 50 °C. It is the simplest and the cheapest method of gels drying. However, the disadvantage of this technique is related to the formation of a liquid meniscus in each pore while solvent evaporates from the surface of the gel. The capillary forces induced by the solvent within the pores generate pressures differences between 100 and 200 MPa [93], which cause an extreme decrease in the final material porosity.

Lyophilization is a drying process based on freezing the solvent present in the pores, followed by its sublimation [94]. However, during solvent freezing, dimensional variations of solvent occur, causing tensions in the gel structure. This can cause fissures or even lead to a complete destruction of the initial geometric gel structure, resulting in a powder as a final product [63]. Therefore, the use of a solvent that has minimum volume variation during freezing is required, coupled with a high vapor pressure to promote sublimation. *Tert*-butanol (2-methyl-2-propanol) is generally used to minimize the effects of volume and structural modification of a cryogel due to its low-density (−3.4 × 10^−4^ g/cm^3^) and low vapor pressure variation (821 Pa) [94] at the freezing point compared to water, −7.5 × 10^−2^ g/cm^3^ and 61 Pa, respectively [95].

The supercritical drying technique is based on increasing both the pressure and temperature of the solvent beyond the critical point to avoid the formation of a vapor-liquid meniscus in the hydrogel pores. Such a technique minimizes gel shrinkage, and consequently the porosity loss due to low capillary forces generated [90,96]. Organic solvents such as acetone [77] are used for drying tannin based gels to produce aerogels at a critical temperature and pressure of 250 °C and 14 MPa, respectively. This is known as the "HOT process" where the drying step is carried out at high temperature conditions [66]. It can also be performed in the presence of CO_2_ [60], which is called the "COLD process" [65,90] at a critical temperature and pressure of 40 °C and 10.4 MPa, respectively. The latter requires the exchange of two solvents due to low solubility of CO_2_ in water. First, water is replaced by ethanol, followed by liquid CO_2_ exchanging.

Usually, aerogels maintain large part of their geometric and nanometric structures, which is associated to lower volume shrinkage. Thus, the initial porosity is largely preserved, and aerogels regularly present low values of bulk density and high values of specific surface area and pore volumes [60,77,89].

**Figure 11:**
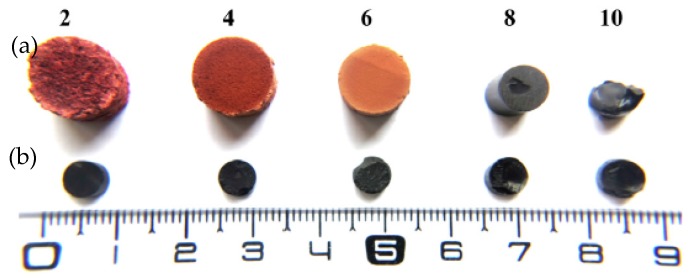
Tannin-formaldehyde gels prepared with surfactant (**a**) top view, and without (**b**) bottom view. Reprinted with permission from reference [98]. Copyright 2011 Elsevier.

In order to avoid capillary tension, gels can be synthesized directly in solvents with surface tension lower than water, such as acetone or ethanol. However, as water is always produced during polycondensation reactions [62], the formation of a vapor-liquid meniscus could not be totally avoided. Thus, surfactants are employed in gels synthesis to reduce the effects of surface tension in xerogels during their drying [61,97,98] (see more details in Table 1). Furthermore, surfactants may also be used as a template to produce ordered porous materials based in a self-assembled micellar system [99]. Xerogels prepared from tannin-formaldehyde with a mass fraction of 25 wt.% and surfactant (Pluronic F-127) (Figure 11), had bulk densities about (0.28-0.65) g/cm^3^ comparable of tannin aerogels [98]. The numbers 2 to 10 in Figure 11 refer to initial pH of tannin-formaldehyde-pluronic solutions, and their highest T_gel_ (~240 min) was found to be at pH 4 and 85 °C [61].

The use of additives, e.g., surfactants, can decrease the capillary stresses during the drying step. Tannin can indeed be used in a wide range of pH (2–10), differently from lignin and phenol (that only work at alkali pHs) or resorcinol (that only works from mildly acid to alkali pHs). The final texture properties of organic porous gels change with several parameters, such as the pH of the initial solution, the mass fraction of solids, the raw materials, the chemicals employed to modify the initial pH of the solution (alkali and acids), the temperature of gelation, and the drying method used. Thus, there is no unique recipe to control the final properties of gels, since each parameter plays a specific role, as explained before. There is an optimal condition for each system, depending on the desired final property. 

A summarized description of organic tannin gels prepared with formaldehyde by different systems, as well as the description of the drying methods used and their physicochemical properties, are reported in Table 1. To produce an organic gel with high specific surface area, it is preferable to choose materials with the highest gelation time (which depends on the pH) and with a mass fraction between intermediate to more diluted (˂20 wt. %). High mesopore volumes are used for tannin gels prepared in the presence of soy, whereas monolithic, unimodal, or bimodal organic gels are readily prepared in the presence of a surfactant such as pluronic.

### 2.4. Hydrogels Formulations under Hydrothermal Conditions 

Hydrothermal carbonization (HTC) is a wet thermochemical conversion process to convert biomass (or wet biomass) into fuels in solid and liquid phases [100]. This technique has been known since 1913, but there has been an increasing interest in it only during the last decade. HTC uses moderate temperature (180−260 °C) and pressure (1−4.7 MPa) to transform biomass precursors into hydrochars with a hydrophilic shell and a hydrophobic core. Recently, the HTC technique has allowed the production of gels made from various precursors, e.g., phloroglucinol with some carbohydrates (glucose, fructose, or xylose) [101], borax and glucose [102], *S*-(2-thienyl)-l-cysteine and 2-thienyl carboxaldehyde [103], d-glucose and ovalbumin [104] and mimosa tannin in ammonia solution [105], without any crosslinker agent (formaldehyde) and at a much lower reaction time (e.g., 24 h). 

**Figure 12:**
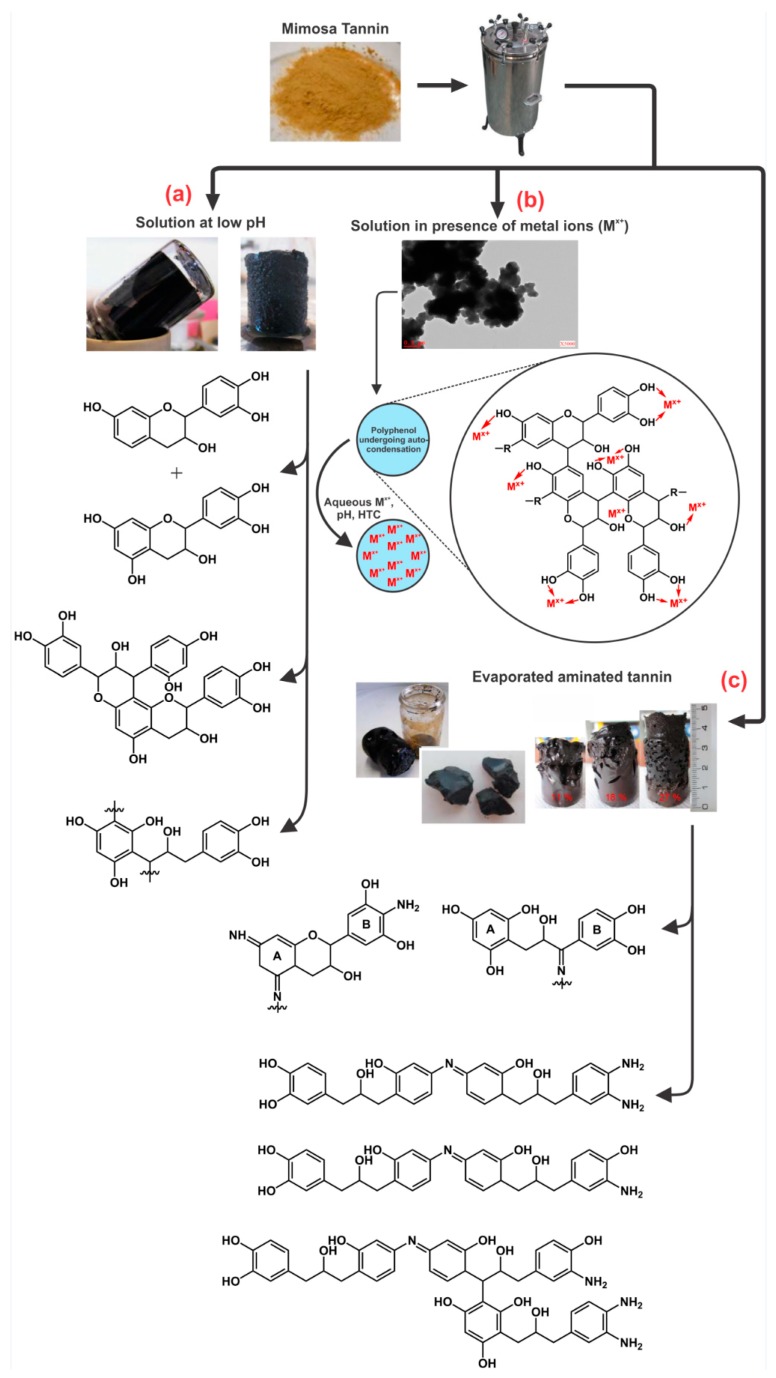
Gels prepared under hydrothermal conditions with aqueous tannin solution at low pH (**a**) (Reprinted with permission from reference [106]. Copyright 2015 Elsevier), with metal salts (**b**) [113], and with aqueous evaporated aminated tannin (**c**) [114].

Figure 12 presents the different organic gels prepared under HTC conditions with: (a) tannin aqueous solution at low pH [106]; (b) tannin aqueous solution in presence of a metal salt; and (c) evaporated aminated tannin. The first gels (Figure 12 a) were elaborated under HTC conditions (180 °C for 24 h) simply with tannin solution in a very acidic environment (pH lower than 3). Three main reactions occurred with acidic tannin solution under heat: (i) the formation of soluble anthocyanidin and catechin [70,107]; (ii) the rearrangement to phlobaphenes, which are insoluble in water and have high molecular weight (Figure 2) [18,108] and; (iii) possibly condensation between the free radical coupling of B-ring catechol units in the presence of atmospheric oxygen [70]. In the same HTC conditions, tannin dissolved with metal salts, especially chromium, lead to a nano-structure powder material, but its microscope structure showed small particles aggregated together in a nodular form, which is typical of a gel structure [94]. This is due to the tannin characteristic of being good metal chelators (Figure 4). Thus, metal ions were chelated with most of hydroxyl groups in tannin-based polymer networks under HTC conditions (Figure 12b). 

Another gel produced under the same hydrothermal conditions as previously mentioned was prepared from aminated tannin. In a typical synthesis, tannin was dissolved in ammonia solution at 28 wt.% and left in a fume hood to evaporate overnight. The solid dried residue recovered was then mixed with water. The final mixture after HTC conditions generated a monolithic material, having the same typical structure of a gel made from tannin and formaldehyde, but in absence of any crosslinker or condensation catalyst [105,109]. The material is composed of very long chains polymerized to an “infinite” three-dimensional network that were not even detected by MALDI-ToF analysis due to their high molecular weight. The mechanism of gel formation is based on selfcondensation and partly dehydrated tannin, mainly through the heterocycle opening [105,109]. The same reactions were also reported during tannin amination under ambient conditions [110]. However, under HTC conditions, the formation of this gel is probably due to the creation of −NH− bridges with the amination of C3’ and C4’ (Figure 2) according to ^13^C NMR analysis, as shown in Figure 12c.

The evaporated aminated tannin gel was also dried accordingly to produce xerogel, cryogel and aerogel at the same conditions as mentioned before with tannin-formaldehyde gels [111]. After HTC, these gels were homogeneous and had monolithic shapes, but they were quite fragile, with some cracks here and there. The mass fraction of evaporated aminated tannin was varied at 11, 18, and 27 wt.% to check any possible physicochemical differences with more concentrated gels. Interestingly, xerogels had great porosity, and the drying method had an important impact on the most diluted gel (11 wt.%). At such concentration, the porosity was improved in this sequence: xerogel, cryogel and aerogel (102, 219, and 295 m^2^/g, respectively). The same trend was also observed for tannin-formaldehyde gels under normal conditions (Figure 10). 

A hydrothermal cryogel made from graphene oxide sheets and tannin was developed by Deng et al. [112]. The mechanism of gel formation was based on the self-assembly of graphene oxide with tannin acting as both reductant and surface functionalization agent. The final monolith with a three-dimensional structure had a mesoporous structure and a good performance for strontium removal due to its important amount of oxygen functional groups (see more details in Section 2.6). A summarized description of organic tannin gels made through HTC synthesis conditions and their final physicochemical properties can be seen in Table 2.

### 2.5. Thermal Treatment of Organic Gels

Thermal treatments are very well-known techniques used to transform organic-derived materials into carbonaceous materials from different precursors. Typically, carbonization and activation are the main methods used for the synthesis of carbon gels. Carbonization is based on heating up a dried organic material in an inert atmosphere (nitrogen, helium or argon) at temperatures between 600 and 1000 °C. At such conditions and at slow heating rates, the volatiles are released in a certain form to avoid the shrinkage of the final material structure [115]. Although it may vary from one precursor to another, the influence of temperature, residence time and heating rate have been well studied. For example, it has been demonstrated that temperatures above 900 °C are suitable for removing most volatile compounds [77], developing the porosity of the tannin gel, improving its micropore volume and surface area, and widening the narrow pores as well. This was indeed confirmed by Szczurek et al. [77] through FTIR analysis of the tannin-formaldehyde gel structure after drying and carbonizing at different temperatures (300–900 °C). At lower temperatures (300–600 °C), the functional groups (mostly oxygenated) were still present in their chemical structure, whereas at higher temperatures (700–900 °C), the same groups were drastically reduced due to their gasification during carbonization. Thus, temperature has a positive influence on the formation of microporosity, surface area and fixed C parameters, but it has a negative influence on carbon yield, average porosity, and functional groups that will be volatilized (e.g., N, H, and O content). 

For specific applications (e.g., energy storage systems), gel materials could have very high porosity and surface area, reaching up to 3000 m^2^/g [116]. Thus, another largely known method for improving the textural properties is the activation method. Different organic precursors can be submitted to an activation process (physical or chemical) before or after carbonization. In physical activation, organic gels are introduced in an oven in an inert atmosphere, but also in the presence of CO_2_ or steam at temperatures between 700 and 900 °C [117]. In chemical activation, the organic gel is in contact with a chemical agent instead such as H_3_PO_4_, KOH, NaOH, ZnCl_2_, etc. at temperatures between 400 and 800 °C, which are considerably below the physical activation temperatures [118]. However, the disadvantage of this process is that the final material must be washed to remove residual chemical agents and possibly inorganic materials formed during this kind of activation [119]. The carbonized or activated carbons have higher specific surface area, higher pore volume, resistance against being attacked in acid and basic solutions, hardness, ignition at low temperature (200–500 °C), hydrophobicity and higher electrical conductivity which can be applied for catalysis, gas/air purification, and in the mining, chemicals, water, food, and pharmaceutical industries [120,121].

**Figure 13:**
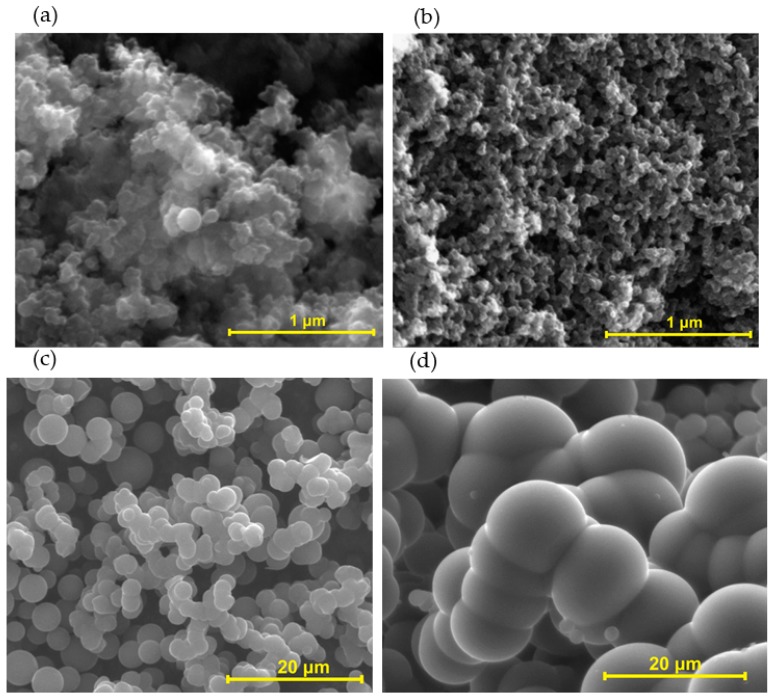
Tannin-formaldehyde carbon aerogels prepared under normal conditions at pH 3.3 (**a**) and pH 8.3 (**b**) (Reprinted with permission from reference [77] Copyright 2011 Elsevier); and hydrothermal carbon gels prepared with tannin solution at pH 2 (**c**) (Reprinted with permission from reference [106] Copyright 2015 Elsevier) and at non-modified pH (4.2) (**d**) [114].

As mentioned before, carbon gels prepared under normal or hydrothermal conditions present textural properties tunable by pH, mass fraction, drying conditions, and others. In the case of gels prepared under normal conditions in presence of formaldehyde, the influence of the pH follows the trend: larger nodules for gels prepared under acid pHs and smaller nodules at alkali pHs (Figure 13a,b). In relation to their textural properties, Table 1 summarizes the main findings. In brief, tannin-formaldehyde gels after carbonization or activation reached the highest values of surface areas (S_BET_) of 1420 m^2^/g and 1810 m^2^/g, respectively. In contrast, the highest S_BET_ values for organic gels were attained at 880 m^2^/g. Thus, the thermal treatment is crucial on the development of porosity, and consequently on the surface area. In addition, these values are comparable to phenol-formaldehyde [122] and resorcinol-formaldehyde [63] aerogels (up to 520 m^2^/g and 695 m^2^/g, respectively) and their carbon derivatives (up to 720 m^2^/g and 580 m^2^/g, respectively).

Table 2 also summarizes the description of tannin carbon gels preparation and their physicochemical and textural properties under HTC conditions. Tannin carbon gels made with different pHs under such conditions had a significant influence on the reaction rate, carbon yield and on the physicochemical characteristics of the final products. Firstly, by modifying the reaction medium, the hydrochar yield increased from 65 wt.% (HTC of tannin solution at non modified pH of 4.2) to 87 wt.% (HTC of tannin solution at pH 1) [106]. The low pH had a positive impact on the final carbon yield and on the lower nodule diameter (Figure 13c,d). The pH of tannin gels made under hydrothermal conditions somehow did not follow the same tendency as for tannin-formaldehyde gels. The gel structure with a lower nodule diameter (at low pH) might have promoted the evolution of volatiles, improving the development of its surface area with almost the same microporous size distribution (800 m^2^/g and 91% micropores) when compared to that with no pH modification (600 m^2^/g and 96% of micropores) [106]. 

Carbon aero-, cryo- and xerogels made from HTC conditions had also similar physicochemical characteristics of gels made from phenol/resorcinol-formaldehyde carbon aerogels [122,123] previously discussed. For example, surface areas of an aerogel made from HTC of evaporated aminated tannin at 27 wt.% and posterior carbonization at 900 °C reached values of up to 900 m^2^/g, having a mixture of well-developed microporosity (54%) as well as wider microporosity and mesoporosity (46%). However, these gels made under HTC were not considered to be monolithic compared to tannin-formaldehyde gel materials. Thus, possible applications for such gels would be in electrochemistry, as the material must be crushed and pressed during the preparation of carbon gel electrodes (see Table 3).

### 2.6. Applications of Organic and Carbon Tannin Gels

Generally, gels have been used for a great deal of applications such as diapers, contact lenses, medical electrodes, breast implants, paints, coatings, adhesives, drug delivery, controlled release of different molecules, adsorbents, and columns for materials separation (chromatography), separation technology (ion exchange), biosensors, catalysis, actuation, and sensing through artificial muscles [129]. However, only a few applications of organic and carbon gels made from tannin are presented in Table 3, according to the available literature. The removal of metals (e.g., Cr^2+^, Cu^2+^, Pb^2+^, and Zn^2+^) was investigated by the usage of tannin-gel type already explained in Section 1.3 (Tannin complexation of metals). They are less porous than gels described in this review, as they did not follow the same procedure of ageing, solvent exchange, and drying methods. Despite their lower porosity, tannin-gels have been reported to be mostly applied as adsorbents for the removal of metal contaminants from water at a concentration above the threshold fixed by environmental regulators. This is because tannin presents phenolic hydroxyl molecules that have a specific affinity to metal ions, and the ability to chelate them. An example of the tannin chelation process is presented in Figure 4. The advantage of using tannin-gel as adsorbent over tannin is that a gel type material is insoluble in water whereas tannin powder is very soluble, and it must be insolubilized or immobilized after the water treatment process. 

It is also noticed that the applications of tannin gels are proposed depending on the gel type, namely aero-, cryo- and xero-gel. For example, two types of organic aerogels made from tannin-soy-formaldehyde and tannin-formaldehyde were tested as thermal insulators. This is because of their very low density (0.05−0.84 g/cm^3^) and high mesopore volumes (up to 2.3 cm^3^/g) [54,60]. The first aerogel presented a thermal conductivity of 0.033 W/mK, which was higher than that of air (0.026 W/mK). The authors reported that even though high porosity was attained, its volume of mesopores was only half of the total pores volume thus, narrower pores and fewer macropores would be necessary to reduce its thermal conductivity [54]. The second aerogel (tannin-formaldehyde), however, presented a thermal conductivity close to the air (0.027 W/mK) due to its low density and a significant fraction of very narrow pores. Additionally, the first aerogels showed a filamentous structure different from the typical “string-of-pearls” found for tannin-formaldehyde gel and thus, the thermal conductivity might have presented a distinct mechanism for both microstructures. This finding demonstrated that depending on the gel type and its physicochemical and textural characteristics (density, pores distribution, surface area, etc.), they can be selected for very specific applications. 

For example, mesoporous materials are very interesting for water treatment because an appropriate pore size distribution is crucial in order to adsorb molecules of different sizes. Indeed, organic molecules (high-size molecule) were found to be successfully adsorbed by materials with larger micropores and mesopores [130]. Bimodal porosity was also found to be interesting for various applications because of the accessibility of ions or compounds to be transported from wider pores to micropores. On the other hand, micropores were found to play an important role in enhancing the specific capacitance. However, micropores with complex pore structure are not very useful for their capacity to store electrical energy. For example, pores lower than 0.7 nm may contribute to improving the specific capacitance, but they are not accessible at a high discharge rate [131]. Thus, the pore structure and pore size distribution must be optimized in order to have materials with high capacitance performances.

Researchers were motivated to explore the electrochemical properties of hydrothermal tannin carbon gels not only because of their great developed porosity, but also due to the enhancement of their functional groups, especially nitrogen and oxygen connected to their surface. This is because supercapacitor performances depend on the surface area accessible to the electrolyte ions, the presence of mesopores, and some heteroatoms, such as oxygen and nitrogen for the improvement of wettability and electronic conductivity [132,133,134]. Indeed, the final evaporated aminated tannin under HTC conditions presented nitrogen compounds in their structure, which had an important influence on the capacity of such materials to store electrical energy. According to Table 3, these materials presented the highest specific capacitance (up to 390 F/g) at scan rate of 2 mV/s [111]. Such values were higher than those reported for biosourced carbon gels in the available literature [126,135,136,137]. This finding is due to the combination of functional groups of oxygen and nitrogen that contributed to the enhancement of pseudo-capacitance through faradaic effects [60,111]. However, the authors highlighted that to achieve even better electrochemical performances at higher scan rates, the mesostructuration within 3 and 13 nm must be created in the structure of the carbon gel for the accessibility of micro- or ultramicro-pores. So, further studies are still needed for an effective application of tannin carbon gels in energy storage.

## 3. Conclusions

The present study reviewed the pertinent literature dedicated to the synthesis, physicochemical and textural properties, and applications of organic and carbon tannin gels. The main findings are outlined below:

- Tannins are the most abundant compounds from biomass after cellulose, hemicellulose, and lignin that can be used in a sustainable biorefinery plant. Among them, condensed polyflavonoid tannins exhibit a complex and heterogeneous structure with highly reactive sites in their structure that lead to condensation, polymerization or rearrangement under acid or alkaline conditions, in the presence of catalysts, metals, sulfite, and especially aldehydes (e.g., formaldehyde), which are efficient precursors for the preparation of solid gels.
- The pH of the solution may also affect the final gels obtained under normal or hydrothermal conditions. Under normal conditions, gels prepared at low pH present high nodules, consequently high clusters, and high porosity, whereas at high pH, gels presented smaller nodules, reduced porosity, and narrow pores. In the case of hydrothermal gels, at low pH, gels attained low spherical nodules with higher porosity compared to bigger spherical particles of lower porosity at high pH. In the last case, smaller nodules favored the evolution of volatile matter during carbonization, and consequently improved the surface area and porosity development.
- The mass fraction of total solids from initial solutions has also an impact on final properties of tannin gels. Basically, very diluted systems lead to more porous gels, whereas high mass fractions tend to produce less porous materials. However, the most porous gels bear more capillary stresses during the drying step, which can be controlled by the addition of a surfactant in the medium.
- During tannin gel synthesis, the drying method defines the porous structure of the organic tannin gel, and consequently its application. Although aero- and cryo-gels are produced from costly techniques, their developed porous structure due to lower shrinkage is desired for several applications, which have not been tested or fully explored as yet (e.g., catalysis, column separation methods, chromatography, and thermal insulation).
- Carbonization and activation conditions play an important role in the porosity development of tannin gels. Optimal conditions of temperature, residence times, heating rate, and physical and chemical agents are suitable for the development of their porosity for specific applications. Thus, thermal treatment parameters conditions must be optimized during the production of tannin carbon gels.
- Tannin gels present interesting physicochemical properties for several applications. However, further studies should be performed to investigate their pore size distribution and the development of mesopores, which are crucial for most of the applications discussed: the removal of contaminants from wastewater, thermal insulation and energy storage. In addition, most studies on the application of tannin gels were based on small-scale tests.
- Thus, tannin organic and carbon gels are based on non-toxic, biosourced, and low-cost materials, which have textural properties (porosity or surface area) that are comparable to synthetic phenolic precursors such as phenol and resorcinol-formaldehyde gels.

## Figures and Tables

**Table 1:** Organic and carbon gels synthesized at normal conditions.

Precursors	Conditions	Main Findings	Ref.
**Hydrogels Prepared Under Normal Conditions** *Organic Tannin Gels*			
Tannin + formaldehyde (TF)	Hydrogels prepared at 85 °C for 120 h; Mass fraction of total solids (MFTS) of 4–40 wt.%; NaOH and p-toluene sulfonic acid were used to change the initial pH of the solution (4.3); pH = 2−10; Supercritical drying in CO_2_	Organic aerogels; S_BET_ = 219–880 m^2^/g; Porosity = 40–97% The highest S_BET_ (880 m^2^/g) and the highest micropore volume (0.28 cm^3^/g) were obtained for a gel prepared with MFTS of 10 wt.% and pH 6. The maximum mesopore volume (1.34 cm^3^/g) and the highest pore volume (21.5 cm^3^/g) were found for samples with a MFTS of 18 and 4 wt.% at pH 8 and 2, respectively. The transparency of gels was associated with the structure of the aquagel: larger nodules at low MFTS (˂ 18 wt.%) and low pH (especially 2) lead to larger clusters and pore volumes (21.5–4.9 cm^3^/g), reducing the transparency of the final gel. By increasing the pH (4–10) and MFTS (22–40 wt.%), the nodules were reduced, and the transparency tended to increase. Aerogels with MFTS greater than 22 wt.% with extremely high pH (10) resulted in a low porous material (0.48–1.01 cm^3^/g). The most diluted samples (4 and 6% of TF resin at pH 6 and 8, respectively) presented the highest shrinkage volumes (~ 87%) during drying step, which was caused by high capillary forces due to their low mechanical properties. These values are comparable to those of aerogels from natural biopolymer, such as cellulose (75 wt.%). The S_BET_ values found for samples with MFTS lower than 20 wt.% were higher than those of any other bioresourced organic aerogel.	[60]
Tannin + formaldehyde + Pluronic (TFP)	Hydrogels prepared at 85 °C for 120 h with Pluronic® F-127, using a mass ratio of T/P of 2; NaOH and p-toluene sulfonic acid were used to change the initial pH of the solution (4.9); pH = 2−10; Subcritical drying in two steps: air and 80 °C	Organic xerogels; S_BET_ = ˂ 1.5 m^2^/g; Porosity = 2–79% The surfactant P was employed as an additive to decrease the surface tension of the aqueous system inner porosity and consequently mitigate the shrinkage during drying step. The system TFP presented a correlation between porosity characteristics (pore volume and centered peak pore size measured through mercury porosimetry) and pH. Thus, pH played a crucial role in pluronic-tannin gels preparation. The xerogels prepared at pH 2–7 presented a bulk density comparable to those of aerogels (0.30–0.58 cm^3^/g). Such a system demonstrated an interesting and low-cost technique for preparing porous gels with tailored porosity on a large scale. The final xerogels presented unimodal (macroporosity) or bimodal (macro-mesoporosity) porosity.	[61]
Tannin + soy + formaldehyde (TSF)	Hydrogels prepared at 85 °C for 120 h; Proportions of T/S resin of 30 and 70 wt.% (on a dry basis), respectively; NaOH in pellets and H_3_PO_4_ (85%) were used to change the initial pH of the solution (8.2); pH 5.5−9; Mass ratio of total solids 14 wt.%; Supercritical drying in CO_2_	Organic aerogels; S_BET_ = 384–478 m^2^/g; Porosity = 84–88% The pH of the initial solution had a significant impact on gelation: the lowest gelation time, which happened at pH 6, produced the highest S_BET_ (478 m^2^/g) and micropore volume (0.15 cm^3^/g). Curiously, this aerogel also presented the highest mesopore volume (2.29 cm^3^/g). A correlation between S_BET_ and gelation time was noticed. The TSF aerogels were based on a filamentous structure, which is comparable to cellulosic and lignocellulosic aerogels. Organic aerogels are natural at 91%, and mesopore volumes (1.72–2.29 cm^3^/g) are among the greatest ever volumes reported for aerogels of natural origin, considering the same density (0.19–0.25 g/cm^3^).	[54]
Tannin + lignin + formaldehyde (TLF)	Hydrogels prepared at 85 °C for 120 h; Mass ratio of T/L of 0.11–1 (on a dry basis); Mass ratio of ((L + T)/F) of 0.83–2.5 (on a dry basis); Initial pH of 10; Supercritical drying in CO_2_	Organic aerogels; S_BET_ = 50–550 m^2^/g; Porosity = 72%–87% The S_BET_ of TLF aerogels was mostly related to mesopores volumes (0.2–1.4 g/cm^3^), since micropores were extremely low (0.01–0.02 cm^3^/g). The highest S_BET_ values were found for TLF gels at mass fractions of T/L=1 and (L + T)/F = 1.25, demonstrating that tannin could be replaced by lignin at 50 wt.%. Besides, there is an optimal quantity of formaldehyde that can be used to produce highly porous aerogels. Organic aerogels were synthesized using up to 71% of natural precursor.	[78]
Tannin + resorcinol + formaldehyde + sodium dodecyl sulfate (TRFSDS)	Preparation in oven: Hydrogels prepared at 85 °C for 72 h (gelation) and 48 h (curing); Preparation in a microwave: 85 °C for 3 h (gelation and curing) and 1–2 h (drying); NaOH and SDS were used as catalyst and surfactant, respectively; Initial pH of 5.5; SDS amount of 5 wt.%; Mass ratio of (T + R)/F of 1.2; MFTS of 25 wt.%; T was used in different proportions (0–100 wt.%) to replace R	Organic xerogels; Porosity = 20–85% The synthesis of tannin xerogels in a microwave oven allowed a reduction in time of 95%, including the time from sol-gel reaction until complete drying. Resorcinol could be replaced by tannin in the production of organic xerogels. However, the use of surfactant is always required because the structure collapses in the presence of large quantities of tannin (>25 wt.%). Materials with high proportion of tannin (75 wt.%) were produced with very small difference of porosity through conventional and microwave synthesis conditions (77% and 62%, respectively).	[124]
*Carbon tannin gels*			
Tannin microspheres (TM)	Microspheres prepared at 80 °C for 1 h stirred at 200–1200 rpm; Mixture of surfactant (SPAN 80) (0–10%) and sunflower oil; Initial pH of 4.3; MFTS of 40 wt.%; Carbonization: 900 °C for 2 h; Heating rate of 2 °C/min	Carbon xerogels; S_BET_ = 7–666 m^2^/g Microspheres were prepared by inverse emulsion polymerization in sunflower oil, followed by subcritical drying and carbonization. Higher stirring speed produced microspheres with low average size due to the shear rate that lead particles (microspheres) at smaller sizes. At the same time, smaller microspheres were produced by increasing the surfactant content and promoting greater contact between oil and the aqueous phase. This process formed small drops of resin that became microspheres after gelation. However, there was a limit amount of surfactant and speed stirring to produce small microspheres of 5 wt.% and 500 rpm, respectively. The mean deviation decreased with the increasing speed stirring (with or without surfactant) and the final microspheres presented narrow micropore size distribution centered at 0.4–0.5 nm.	[125]
Tannin + formaldehyde (TF)	Hydrogels prepared at 85 °C for 120 h; pH from 3.3 to 8.3; Supercritical drying in acetone; Carbonization: 900 °C for 2 h; Heating rate of 5 °C/min	Carbon aerogels; S_BET_ = 580–720 m^2^/g; Porosity = 75–99% Carbon aerogels based on tannin-formaldehyde solutions were prepared in a broad pH range. The microstructure of gels presented the typical nodular shape of phenolic aerogels, where their size was determined by the initial pH of the solution. The polymerization reaction was performed with methylene or methylol bridges, as showed by ^13^C NMR analysis. The final material presented high mesopore fraction (57–78%). The synthesized TF aerogels were five times cheaper than resorcinol-formaldehyde aerogels.	[77]
Tannin + resorcinol + formaldehyde (TRF)	Hydrogels prepared at 50 °C for 10–160 min (depending on gelation time); Acetic acid or orthophosphoric acid were used to change the initial pH of the solution (8.15); pH = 2−8; Freeze-drying; Carbonization: 900 °C for 2 h; Heating rate of 5 °C/min	Carbon cryogels; S_BET_ = 30–650 m^2^/g; Porosity = 32–80% Low cost cryogels were produced by replacing two third of resorcinol by tannin, which resulted in a reduction of the final cost of the gel by a factor of 2. Also, freeze-drying represents a low-cost drying method compared to supercritical drying. The S_BET_ and porosity decreased by increasing pH. Furthermore, S_BET_ values were comparable to resorcinol-formaldehyde cryogels and phenol-formaldehyde aerogels, which are gel materials based on more expensive precursors.	[53]
Tannin + formaldehyde (TF)	Hydrogels prepared at 85 °C for 120 h; Acetic acid or NaOH were used to change the initial pH of the solution (4.3); pH = 3.3−7.3; Freeze-drying; Carbonization: 900 °C for 2 h; Heating rate of 5 °C/min	Carbon cryogels; S_BET_ = 399–1420 m^2^/g; Porosity = 94–96% Carbon cryogels presented high pore volumes, mostly distributed in macro- and micropores sizes. These materials also presented very low bulk densities (0.078–0.101 g/cm^3^), mostly composed by macropores (0.13–0.55 cm^3^/g). Materials prepared at pH higher than 6 presented higher S_BET_ (1228–1420 m^2^/g) and higher micropore volumes. The electrochemical performances of those materials were tested, and the main results are presented in Table 3.	[126]
Tannin + pluronic + formaldehyde (TPF)	Hydrogels prepared at 85 °C for 120 h; pH from 2 to 10; Subcritical drying in two steps: air and 80 °C; Mass ratio of T/P of 0.5–2; Carbonization: 900 °C for 2 h; Heating rate of 5 °C/min	Carbon xerogels; S_BET_ =5–877 m^2^/g; Porosity = 46%–86% The use of pluronic allowed the synthesis of the first carbon tannin based xerogels presenting density values in a range of 0.28–0.33 g/cm^3^, which are comparable to those of carbon aerogels derived from expensive precursors. Those densities are related to samples prepared at pH lower than 7 and low amounts of pluronic (Mass ratio of T/P of 2). Narrow bimodal macro-microporous or meso-microporous xerogels were produced as a function of pH and surfactant. For lower pluronic concentrations, the micropore size distribution remained centered at 0.5 nm, while the macroporosity was shifted as a function of both pluronic and pH. By increasing the amount of pluronic and keeping the same pH, the porosity changed from a macro-microporous structure to a meso-microporous structure. The final structure was highly porous with narrow non-ordered porosity.	[98]
Tannin (T) (autocondensation)	Tannin was added to solutions of Na_2_SiO_3_ (35 wt.%); Mass ratio of SiO_2_/T of 0.3–1.04; NaOH (2 mol/L) or HF (40 wt. %) were used as etching agents; Curing and ageing were performed at ambient temperature for 3 h; Supercritical drying in CO_2_; Carbonization: 900 °C for 2 h; Heating rate of 5 °C/min	Carbon aerogels; S_BET_ NaOH = 451–709 m^2^/g; S_BET_ HF= 542–783 m^2^/g; Porosity = 67%–82% Porous carbon gels were prepared with no formaldehyde addition. Na_2_SiO_3_ was used as catalyst for auto-condensation and as template for porosity formation. The final porous properties were controlled by silica/tannin ratio, while NaOH or HF were used as etching agents. XPS and Raman analyses showed the non-ordered aromatic structure of tannin-based carbon aerogels. Carbon aerogels washed with HF solutions presented better porosity (71%–82%) compared to NaOH (67%–75%). Samples prepared at low SiO_2_/T mass ratio (0.30–0.75) had no significant differences. However, the highest SiO_2_/T mass ratio (1.04) presented the lowest density, and consequently the highest porosity. By increasing the quantity of silica in the reactional medium, there was a decrease in the microporosity associated to S_BET_, whereas the highest values were found for SiO_2_/T = 0.45. Mesopores were better developed at SiO_2_/T mass fraction of 0.75 (1.71 cm^3^/g for samples etched with HF).	[127]
Tannin + sodium dodecyl sulfate + formaldehyde (TSDSF)	Hydrogels prepared at 85 °C for 72 h; NaOH and sodium dodecyl sulfate (SDS) were used as catalyst and surfactant, respectively; Initial pH of 5.5, SDS amount of 5–20 wt.% and T/F mass ratio of 0.6–2.6; MFTS of 25 wt.%; Supercritical drying in CO_2_; Carbonization: 900 °C for 2 h; Heating rate of 5 °C/min	Carbon xerogels; Porosity = 25%–78% There was an optimal amount of surfactant (10 wt.%) for producing highly porous carbon xerogels (density of 0.34 g/cm^3^ and porosity of 78%). However, when using 10 wt.% of surfactant and increasing the T/F mass ratio, the pore volumes increased, and the average pore size decreased. Also, the concentration of formaldehyde was important for attaining materials with greater porosity, since an ideal proportion of crosslinker promotes the creation of links between surrounding clusters.	[97]
Tannin + formaldehyde (TF)	Hydrogels prepared at 85 °C for 120 h; pH of 2, 5 and 8; Activation with NaOH or KOH; Mass ratio of NaOH/gel of 0.045, 0.181 and 0.724 (on a dry basis); Mass ratio of KOH/gel of 0.063, 0.253 and 1.013 (on a dry basis); Activation: 750 °C for 1 h; Heating rate of 5 °C/min	Activated carbon xerogels (ACX); S_BET_ = 50–1810 m^2^/g The exchange step was performed with alkali solutions at different concentrations. Higher concentrations of alkali (NaOH or KOH) induced the development of surface area. In addition, the microstructure of ACX changed from nodular to microcellular carbon foam and, the effect of the pH was not meaningful in these different structures. A smaller dispersion of particle sizes and high surface areas related to micropores volumes were obtained using smaller mass ratio of alkalis (0.045–1.013), compared to usual values in the literature (>1). Probably, the soft structure of gels allowed the alkali solution to interpenetrate in its three-dimensional structure, causing greater alkali diffusion and favoring the formation of higher porosity.	[118]

**Table 2:** Organic and carbon tannin gels synthesized under hydrothermal conditions.

Precursors	Conditions	Main Findings	Ref.
**Hydrogels Prepared Under Hydrothermal Conditions** *Organic and Carbon Hydrothermal Tannin Gels*			
Evaporated aminated tannin (EAT)	Hydrothermal gel: Amination at room temperature followed by evaporation and HTC of the solid material in water at 180 °C for 24 h; Subcritical drying in two steps: air and 80 °C; Carbonization: 900 °C for 3 h; Heating rate at 1 °C/min.	Xerogel: S_BET_ = 32 m^2^/g This amination process under HTC conditions lead to a gel without any crosslinker agent. The mechanism was based on autocondensation and partly dehydrated tannin mainly through the heterocycle opening. Materials also had high percentage of nitrogen (up to 3.4%). Carbon xerogel: S_BET_ = 500 m^2^/g The synthesized carbon gel had a porous structure comprised of a mixture of wider microporosity and mesoporosity (Isotherms Type I and IV). Materials had high percentage of nitrogen (up to 2.9%) and a micropore/total pore volume ratio of 72%. XPS analysis showed several forms of nitrogen such as pyridinic (N-6) and neutral amines in the hydrothermal xerogel, whereas pyridic (N-6), pyrrolic (N-5) and oxidized (N-X) (stable forms of nitrogen) were found in its carbon form.	[105]
Evaporated aminated tannin (EAT)	Hydrothermal gel: Amination at room temperature followed by evaporation and HTC of the solid material in water at 180–220 °C for 24 h; Subcritical drying in two steps: air and 80 °C; Carbonization: 900 °C for 3 h; Heating rate at 1 °C/min.	Xerogel: S_BET_ = 64−102 m^2^/g A monolithic gel was obtained under HTC at temperatures from 180 to 220 °C. Not many differences in relation to the physicochemical properties were observed in this range of temperature. These gels were nitrogen rich materials (up to 4.3%). Carbon xerogel: S_BET_ = 311−552 m^2^/g Higher HTC temperatures lead to lower surface areas (~ 300 m^2^/g) due to the formation of bigger and less porous spherical nodules. After carbonization, materials still had a high percentage of nitrogen (up to 2.9%). N-doped materials were successfully synthesized for electrochemical devices. These results were presented and discussed in Table 3.	[109]
Evaporated aminated tannin (EAT)	Hydrothermal gel: Amination at room temperature followed by evaporation and HTC of the solid material at mass fractions of 11, 18 and 27% in water at 180 °C for 24 h. The final gels were first soaked in ethanol for 3 days, and then subcritically (air and 80 °C), supercritically (CO_2_), and freeze-dried; Carbonization: 900 °C for 3 h; Heating rate at 1 °C/min.	Xerogel, aerogel and cryogel: S_BET_ = 102−295 m^2^/g For the most diluted gels (11 wt.%), the porosity improved in this sequence: xerogel, cryogel and aerogel. For the least diluted gels, similar surface areas were obtained at any kind of drying method. The mechanism of gel formation was described in Section 2.4. Carbon xerogel, aerogel and cryogel: S_BET_ = 496−860 m^2^/g Unlike organic gels, carbon gels prepared with the highest mass fraction (27 wt.%) had the highest surface area, up to 900 m^2^/g. It was then noticed that the most porous organic gels do not lead to carbon gels with the highest surface areas.	[111]
Tannin (T)	Hydrothermal gel: Tannin aqueous solution under HTC conditions at 180 °C for 24 h; pTSA (para-toluenosulfonic acid) was used to change the pH from 4.2 to 1; Subcritical drying in two steps: air and 80 °C; Carbonization: 900 °C for 3 h; Heating rate at 1 °C/min.	Xerogel: S_BET_ = 1.25 m^2^/g Monolith material was obtained by reducing pH, resulting in a positive impact on the hydrochar yield. However, a very low surface area was obtained. Carbon xerogel: S_BET_ = 796 m^2^/g Monolithic materials having spherical nodules at different diameters were obtained: 2.9 µm at pH 2; and 4.6 µm at pH 4.2. This trend was different from phenolic gels prepared with tannin-formaldehyde. The surface area of 796 m^2^/g was attained for the material prepared at lowest pH. Its higher microsphere nodule diameter might have promoted the gasification of low molecular weight gases, which enhanced the development of their surface area.	[106]
Evaporated aminated (pine) tannin (EAT)	Hydrothermal gel: Amination at room temperature followed by evaporation and HTC of the solid material in water at 180 °C for 24 h; Subcritical drying in two steps: air and 80 °C; Carbonization: 900 °C for 3 h; Heating rate at 1 °C/min.	Xerogel: Compacted monoliths having homogeneous spherical particles typical of gels. Carbon xerogel: S_BET_ = 485 m^2^/g Micro-mesoporous carbon gels exhibited high surface area with high nitrogen and oxygen functionalized groups, which were applied as electrodes for electrochemical devices. These results were described in Table 3.	[128]
Bayberry tannin and graphene oxide (GO)	Hydrothermal gel: A mixture of graphene oxide and tannin at different mass ratios (1:0–1.5); Sonication for 30 min and HTC at 120 °C for 12 h.; Gels were soaked in HNO_3_ solution for 4 days; Freeze-drying.	Hydrothermal cryogel: S_BET_ = 75 m^2^/g The monolith had a three-dimensional structure, consisting of crosslinked GO sheets, GO sheets, and tannin. The gels showed a type III isotherm characteristic of mesoporous materials, having surface area up to 75 m^2^/g. Its application as adsorbent to remove Sr^2+^ from water is described in Table 3	[112]

**Table 3:** Applications of organic and carbon gels made from tannin.

Application	Type of Gel	Testing Conditions	Main Findings	References
*Organic tannin gels*				
Thermal insulator	Aerogel	Thermal conductivity of soy-tannin-formaldehyde in a cylindrical shape was performed at room temperature	Tannin gel conductivity is higher than that of air (0.033 W/mK compared to 0.026 W/mK). The presence of narrow mesopores and fewer macropores would be required to improve their performance as thermal insulators.	[54]
Thermal insulator	Aerogel	Thermal conductivity of tannin-formaldehyde in a cylindrical shape was performed at room temperature	Tannin gel conductivity is close to that of air (0.027 W/mK). Low thermal conductivity was reported for gels with low density and very narrow pores.	[60]
*Adsorbent for water treatment*				
Metal Pb^2+^	Tannin-gel	Synthetic effluent 1000 mg/L, batch tests; ratio metal: adsorbent 0.1 g: 100 mL; Equilibrium time 5 h, pH = 1−7, T = 20 °C	Tannin-gel behavior as ionic exchanger: two Na^+^ ions exchanged by one Pb^2+^ ion. Pb removal efficiency increased from 58 to 115 mg Pb/g with increased pH from 3 to 4.2, respectively.	[84]
Metal Cr^6+^	Tannin-gel	Synthetic effluent 1000 mg/L, batch tests; ratio metal: adsorbent 2.5 g:100 mL; Equilibrium time 30 min, pH = 0.85–5, T = 30 °C	Adsorption mechanism consists of four steps: 1) Esterification of chromate with tannin molecules; 2) Reduction of Cr^6+^ into Cr^3+^; 3) Formation of carboxyl groups by tannin oxidation; and 4) Ion exchange of Cr^3+^ with carboxyl and hydroxyl groups. Maximum adsorption capacity: 287 mgCr/g at pH 2.	[83]
Metal Cr^6+^	Tannin-gel	Synthetic effluent 500–5000 mg/L, batch tests; ratio metal: adsorbent 0.2 g:100 mL; Equilibrium time 8 h, pH = 1–12, T = 25–45 °C	Cr adsorption reached the maximum value of 488 mg/g at pH 1 (25 °C). The mechanism of Cr adsorption is based on: 1) Cr(IV) adsorption by phenolic groups through chromate esterification with tannin-gel surface; 2) Cr(VI) reduction to Cr(III); 3) Carboxylate group formation due to tannin-gel oxidation; 4) Cr(III) retention on tannin-gel surface; and finally 5) Cr adsorption through hydroxyl and carboxyl groups.	[138]
Metal Cu^2+^	Tannin-gel	Synthetic effluent 10−150 mg/L, batch tests; ratio metal: adsorbent 0.1 g:100 mL; Equilibrium time 3 h, pH = 2−5, T = 25 °C	Adsorption mechanism is a result of ion exchange or complexation between Cu^2+^ ions and phenolic groups present on tannin-gel surface. Adsorption decreases at lower pH due to ion exchange equilibrium. Maximum adsorption capacity: 44 mgCu/g at pH 5.	[87]
Metal Zn^2+^	Tannin-gel	Synthetic effluent, batch tests; Equilibrium time 2 weeks, pH = 7, T = 20 °C	Tannin-gel made from lab-extracted Pine tannin presented the best performance for Zn removal. Maximum adsorption capacity: 65 mgZn/g	[88]
Metal Ni^2+^	Tannin-gel	Synthetic and real effluent (synthetic and river water) 50−200 mg/L, batch tests; ratio metal: adsorbent 0.25−1 g: 100 mL; Equilibrium time 35 min, pH = 2–7, T = 23 °C	The adsorption of Ni ions took place in a homogeneous tannin gel surface (monolayer adsorption). Maximum adsorption capacity: 250 mgNi/g at pH 5	[139]
Metal Sr^2+^	Hydrothermal cryogel	Synthetic effluent 10−150 mg/L, batch tests; ratio metal: adsorbent 0.02 g:100 mL; Equilibrium time 10 h, pH = 9, T = 25 °C	Graphene oxide-tannin gel prepared under HTC conditions showed excellent adsorption performance for the removal of Sr^2+^ (68 mgSr/g). Surface chemical analysis showed that Sr^2+^ was largely dependent on oxygen functional groups, pH, salinity and ionic strength.	[112]
Rare metal V	Tannin-gel	Synthetic effluent 0.2 mM, batch tests; ratio metal: adsorbent 0.02 g:100 mL; Equilibrium time 1 h, pH = 1–8, T = 30 °C	V was efficiently adsorbed from different solutions: VOCl_2_ and NH_4_VO_3_. Stable compounds were formed between VO^2+^ (acid character) and catechol and pyrogallol (alkali behavior). V adsorption from NH_4_VO_3_ was based on adsorption of H_3_VO_4_ (pH = 3.75) and reduction of VO^2+^ to VO_3_^-^ at pH 6.	[140]
Rare metal Au	Tannin-gel	Synthetic effluent 10 mg/L, batch tests; ratio metal: adsorbent 0.01 g:100 mL; pH = 2−3.8, T = 20 °C	Adsorption of Au took place through the reduction of trivalent Au ions into metallic Au as well as oxidation of hydroxyl groups present in tannin-gel to carbonyl groups. Maximum adsorption capacity: 8000 mgAu/g	[85]
Rare metal Au	Tannin-gel	Synthetic effluent 0.1 mM, column tests; Flow bed at 150 and 300 mL/h; pH = 2–6	98.5% of Au from HAuCl_4_ was adsorbed at pH 6. The mechanism of Au adsorption was based on: 1) Ligand exchange: AuCl_4_^-^ with hydroxylphenyl groups present in the tannin-gel; 2) Reduction of Au(III) to Au(0); and 3) Adsorption of Au(0).	[141]
Rare metal Pd(II)	Tannin-gel	Synthetic effluent 10 mg/L, batch tests; ratio metal: adsorbent 0.04 g:100 mL; pH = 1.3−2.5, T = 25 °C	Adsorption of Pd(II) was based on the inner sphere redox reaction: Pd(II) ions were adsorbed as metallic Pd; hydroxyl groups were oxidized; and a ligand-substitute Pd(II) tannin inner sphere complex was formed.	[86]
Rare metal Pd(II)	Aminated tannin-gel freeze-dried	Synthetic effluent 100 mg/L, batch tests; ratio metal: adsorbent 0.1 g:100 mL; Acidic medium, T = 25 °C	Adsorption of Pd(II) was due to metal ions complexation and/or electrostatic interaction. Also, acidic metal ions had high affinity towards amine basic groups. Maximum adsorption capacity: 80 mgPd/g.	[142]
Rare metals Pd and Pt	Aminated tannin-gel freeze-dried	Synthetic effluent 0.001 M, batch tests, single and multiple metal solutions; ratio metal: adsorbent 0.1 g:100 mL; Equilibrium time 20 h, pH = 0–5, T = 25 °C	The adsorption of Pd and Pt on tannin-gel surface increased with increasing pH and temperature, and with decreasing chloride ion concentration. The amino groups presented in tannin-gel formed stable complex with metal ions but the adsorbability of Pd(II) was much higher than Pt(IV). Interesting that aminated tannin gel adsorbed mostly Pd(II) from mixed solutions even though it had good adsorbability for Pt(IV) from single metal solution	[143]
Rare metals Au, Pd, Pt and Rh	Tannin-gel	Synthetic effluent 1 mmol/L, batch tests, single and multiple solutions; ratio metal: adsorbent 0.1 g:100 mL; pH = 1, T = 25 °C	The predominant species of each metal were adsorbed by controlling the pH (equal to 1) as well as the redox potential differences between metals and tannin-gel. Au was selectively adsorbed and reduced because its redox potential was higher than that of tannin-gel. However, the other precious metals had much lower redox potential than that of tannin-gel.	[144]
Metals: Au(III), Pd(II), Pt(IV), Cu(II), Fe(III), Ni(II), Zn(II)	Tannin-gel	Synthetic and real effluent, batch and column tests, single and multiple metal solutions; ratio metal: adsorbent 0.1–2 g:100 mL; Flow bed at 5 mL/h; Equilibrium time 12 h, acidic pH, T = 30 °C	Rare metals were efficiently adsorbed through both batch and column tests. Tannin-gel selectively adsorbed Au(III), Pd(II) and Pt(IV) over other metals: Cu(II), Fe(III), Ni(II) and Zn(II). The mechanism of adsorption of precious metals was the combination of ion exchange, electrostatic interaction and coordination with thiocarbonyl group. Au(III) was reduced to elemental Au through abundant polyphenolic groups on tannin molecule. Tannin-gel was regenerated under acid solution up to five times.	[145]
Boron (B)	Aminated tannin-gel freeze-dried	Synthetic effluent 200 mg/L, batch tests; ratio contaminant: adsorbent 0.5 g:100 mL; Equilibrium time 20 h, pH = 8.8, T = 30 °C	Aminated and non-aminated gels efficiently adsorbed B at pH > 7. The adsorption of B took place through the chelate formation of tetrahydroxyborate ion and the hydroxyl and amino groups presented in tannin-gels. The adsorption capacity of the aminated cryogel was higher than that of non-aminated one due to the stable bonds between boron and nitrogen from amino groups.	[146]
Phosphate (P)	Tannin-gel freeze-dried	Synthetic effluent 100 mg/L, batch tests; ratio contaminant: adsorbent 0.5 g:100 mL; pH = 2−12, T = 25 °C	The gel impregnated with iron and oxidized with nitric acid showed adsorption selectivity for phosphate. The adsorption process was independent of the pH (from 3 to 12). Maximum adsorption capacity: 31 mgP/gFe	[147]
Organic MB	Tannin-gel	Synthetic effluent, batch tests; Equilibrium time 2 weeks, pH = 7, T = 20 °C	Tannin-gel made from lab-extracted Pine tannin presented the best performance for MB removal. Maximum adsorption capacity: 432 mgMB/g	[88]
Organic MB	Tannin-gel	Synthetic effluent 1000 mg/L, batch tests; Equilibrium time 15 days; pH = 4−10, T = 20 °C	Adsorption of MB was improved by increasing pH, probably because the dye appeared with a higher cationic degree and thus, it enhanced electrostatic interactions. Maximum adsorption capacity: 483 mgMB/g	[148]
Organic BR	Tannin-gel	Synthetic effluent 40 mg/L, batch tests; ratio contaminant: adsorbent 0.04 g:100 mL; Equilibrium time 1.5 h, pH = 2–8, T = 28 °C	Good adsorption of BR due to the presence of functional groups on tannin-gel structure: phenolic, carboxylic, alcoholic, ether and aromatic rings. Maximum adsorption capacity: 45 mgBR/g	[149]
Organic CTAB	Tannin-gel	Synthetic effluent, batch tests; Equilibrium time 2 weeks, pH = 7, T = 20 °C	Tannin-gel made from lab-extracted Pine tannin presented the best performance for CTAB removal. Maximum adsorption capacity: 773 mgCTAB/g	[88]
Benzene and toluene	Tannin-gel	Synthetic effluents 1% sol., batch tests; Equilibrium time 1 h; ratio contaminant: adsorbent 0.1 g:100 mL; pH = 2–8.6, T = 60 °C	The removal of toluene was more effective than benzene probably because of the interactions between the methyl groups on toluene and the OH groups on tannin gel. Results show up to 99% removal of toluene and benzene after 30 min batch tests.	[150]
*Carbon tannin gels*				
Thermal insulator	Carbon xerogel	Thermal conductivity of tannin-formaldehyde-surfactant in a cylindrical shape was performed at room temperature	Carbon tannin gel conductivity is higher than that of air (0.039 W/mK compared to 0.026 W/mK). The presence of narrow mesopores would be required to improve its performance as thermal insulator.	[97]
*Electrochemistry*				
Supercapacitor	Carbon cryogel	Supercapacitor device based on a three-electrode cell configuration with an aqueous acid electrolyte (4 M H_2_SO_4_)	Carbon cryogels prepared at pH higher than 6 had low density and high surface areas (up to 1200 m^2^/g). Thus, such materials as electrodes for supercapacitor reached capacitances as high as 100 F/g (scan rate 2 mV/s). In addition to mesopores, ultra- and supermicropores played an important role on their performance as electrodes for supercapacitor.	[126]
Supercapacitor	Hydrothermal carbon aero-, cryo- and xerogel	Supercapacitor device based on a three-electrode cell configuration with an aqueous acid electrolyte (4 M H_2_SO_4_)	Carbon aerogel, cryogel, and xerogel prepared from HTC of evaporated aminated tannin (MFTS of 27 wt.%) reached surfaces areas of 860, 754, and 585 m^2^/g and specific capacitances of 362, 387, and 330 F/g (scan rate 2 mV/s), respectively. The presence of nitrogen (2−3 wt.%) and oxygen (17−18 wt.%) functional groups played an important role on their performance for electrical energy storage, especially through pseudo-capacitance. However, mesostructuration within 3−13 nm should be created to improve the capacitance reduction at a higher scan rate.	[111]
Supercapacitor	Hydrothermal carbon xerogel	Supercapacitor device based on a three-electrode cell configuration with an aqueous acid electrolyte (1 M H_2_SO_4_)	Hydrothermal carbon xerogel made from evaporated aminated Pine tannin reached a surface area and a specific capacitance of 485 m^2^/g and 253 F/g (scan rate 0.5 mV/s), respectively. The material presented high concentration of nitrogen and oxygen functional groups (6 mmol/g) that played an important role on their performance as electrodes for a supercapacitor.	[128]

MB: Methylene blue; BR: Brilliant red; CTAB: Cetyltrimethylammonium bromide.

## References

[1] Arbenz A., Avérous L. (2015). Chemical modification of tannins to elaborate aromatic biobased macromolecular architectures. Green Chem..

[2] Haslam E. (1989). Plant. Polyphenols: Vegetable Tannins Revisited.

[3] De Hoyos-Martínez P.L., Merle J., Labidi J., Charrier-El Bouhtoury F. (2019). Tannins extraction: A key point for their valorization and cleaner production. J. Clean. Prod..

[4] Pizzi A. (1980). Tannin-based adhesives. J. Macromol. Sci. Polymer Rev..

[5] Pizzi A. (2006). Recent developments in eco-efficient bio-based adhesives for wood bonding: Opportunities and issues. J. Adhes. Sci. Technol..

[6] Roux D.G., Paulus E. (1961). Condensed tannins. 8. The isolation and distribution of interrelated heartwood components of *Schinopsis* spp.. Biochem. J..

[7] Drewes S., Roux D. (1963). Condensed tannins. 15. Interrelationships of flavonoid components in wattle-bark extract. Biochem. J..

[8] Abdalla S., Pizzi A., Ayed N., Charrier F., Bahabri F., Ganash A. (2014). MALDI-TOF and ^13^C NMR analysis of Tunisian *Zizyphus jujuba* root bark tannins. Ind. Crops Prod..

[9] Drovou S., Pizzi A., Lacoste C., Zhang J., Abdulla S., El-Marzouki F.M. (2015). Flavonoid tannins linked to long carbohydrate chains—MALDI-TOF analysis of the tannin extract of the African locust bean shells. Ind. Crops Prod..

[10] Roux D.G., Ferreira D., Hundt H.K.L., Malan E. (1975). Structure, stereochemistry, and reactivity of natural condensed tannins as basis for their extended industrial application. Appl. Polym. Symp..

[11] Roux D.G. (1972). Recent advances in the chemistry and chemical utilization of the natural condensed tannins. Phytochemistry.

[12] Roux D.G. (1965). Modern Applications of Mimosa Extract.

[13] Pizzi A. (1994). Advanced Wood Adhesives Technology.

[14] Pizzi A., Pizzi A., Mittal K.L. (2003). Natural Phenolic Adhesives 1: Tannin. Handbook of Adhesive Technology.

[15] Christiansen A.W., Gillespie R.H., Forest Products Laboratory (1986). Wood Adhesives in 1985: Status and Needs.

[16] Pizzi A., Stephanou A. (1993). Comparative and differential behaviour of pine vs. pecan nut tannin adhesives for particleboard. Holzforsch. Holzverwert..

[17] McGraw G.W., Rials T.G., Steynberg J.P., Hemingway R.W., Hemingway R.W., Laks P.E. (1992). Chemistry of Pecan Tannins and Analysis of Cure of Pecan Tannin-Based Cold-Setting Adhesives with a DMA ‘Micro-Beam’ Test. Plant Polyphenols. Basic Life Sciences.

[18] Sealy-Fisher V.J., Pizzi A. (1992). Increased pine tannins extraction and wood adhesives development by phlobaphenes minimization. Holz. Als. Roh. -Werkst..

[19] Roux D.G. (1965). Wattle Tannin and Mimosa Extract.

[20] Pizzi A. (1979). Sulfited tannins for exterior wood adhesives. Colloid Polym. Sci..

[21] Ohara S., Hemingway R.W. (1991). Condensed tannins: The formation of a diarylpropanol-catechinic acid dimer from base-catalyzed reactions of (+)-catechin. J. Wood Chem. Technol..

[22] Pizzi A. (1982). Pine tannin adhesives for particleboard. Holz Als Roh- Werkst..

[23] Pizzi A., von Leyser E.P., Valenzuela J., Clark J.G. (1993). The chemistry and development of pine tannin adhesives for exterior particleboard. Holzforschung.

[24] Valenzuela J., von Leyser E., Pizzi A., Westermeyer C., Gorrini B. (2012). Industrial production of pine tannin-bonded particleboard and MDF. Eur. J. Wood Wood Prod..

[25] Pizzi A., Valenezuela J., Westermeyer C. (1994). Low formaldehyde emission, fast pressing, pine and pecan tannin adhesives for exterior particleboard. Holz Als Roh- Werkst..

[26] Pizzi A., Stephanou A. (1994). Fast vs. slow-reacting non-modified tannin extracts for exterior particleboard adhesives. Holz. Als. Roh. -Werkst..

[27] Meikleham N., Pizzi A., Stephanou A. (1994). Induced accelerated autocondensation of polyflavonoid tannins for phenolic polycondensates. I. ^13^C-NMR, ^29^Si-NMR, X-ray, and polarimetry studies and mechanism. J. Appl. Polym. Sci..

[28] Slabbert N., Hemingway R.W., Laks P.E. (1992). Complexation of condensed tannins with metal ions. Plant Polyphenols: Biogenesis, Chemical Properties, and Significance.

[29] Tondi G., Oo C.W., Pizzi A., Trosa A., Thevenon M.F. (2009). Metal adsorption of tannin based rigid foams. Ind. Crops Prod..

[30] Oo C.W., Kassim M.J., Pizzi A. (2009). Characterization and performance of *Rhizophora apiculata* mangrove polyflavonoid tannins in the adsorption of copper (II) and lead (II). Ind. Crops Prod..

[31] Pizzi A. (2019). Tannins: Prospectives and actual industrial applications. Biomolecules.

[32] Pizzi A., Cameron F.A., Eaton N.J. (1985). The tridimensional structure of polyflavonoid tannins by conformational analysis. J. Macromol. Sci. Part. - Chem..

[33] Thébault M., Pizzi A., Essawy H.A., Barhoum A., Van Assche G. (2015). Isocyanate free condensed tannin-based polyurethanes. Eur. Polym. J..

[34] Pizzi A. (1979). Tannin-based polyurethane adhesives. J. Appl. Polym. Sci..

[35] Pizzi A. (1978). Tannin-formaldehyde exterior wood adhesives through flavonoid B-ring cross linking. J. Appl. Polym. Sci..

[36] Pizzi A., Stephanou A. (1994). A ^13^C NMR study of polyflavonoid tannin adhesive intermediates. II. Colloidal state reactions. J. Appl. Polym. Sci..

[37] Shirmohammadli Y., Efhamisisi D., Pizzi A. (2018). Tannins as a sustainable raw material for green chemistry: A review. Ind. Crops Prod..

[38] Saayman H., Roux D. (1965). The origins of tannins and flavonoids in black-wattle barks and heartwoods, and their associated ‘non-tannin’ components. Biochem. J..

[39] Li X., Basso M.C., Braghiroli F.L., Fierro V., Pizzi A., Celzard A. (2012). Tailoring the structure of cellular vitreous carbon foams. Carbon.

[40] Lacoste C., Basso M.C., Pizzi A., Laborie M.-P., Celzard A., Fierro V. (2013). Pine tannin-based rigid foams: Mechanical and thermal properties. Ind. Crops Prod..

[41] Lacoste C., Basso M.-C., Pizzi A., Celzard A., Ella Ebang E., Gallon N., Charrier B. (2015). Pine (*P. pinaster*) and quebracho (*S. lorentzii*) tannin-based foams as green acoustic absorbers. Ind. Crops Prod..

[42] Mitsunaga T., Doi T., Kondo Y., Abe I. (1998). Color development of proanthocyanidins in vanillin-hydrochloric acid reaction. J. Wood Sci..

[43] Clark-Lewis J.W., Roux D.G. (1959). Natural occurrence of enantiomorphous leucoanthocyanidian: (+)-mollisacacidin (gleditsin) and quebracho(–)-leucofisetinidin. J. Chem. Soc..

[44] Navarrete P., Pizzi A., Pasch H., Rode K., Delmotte L. (2010). MALDI-TOF and ^13^C NMR characterization of maritime pine industrial tannin extract. Ind. Crops Prod..

[45] Abdalla S., Pizzi A., Ayed N., Charrier-El Bouthoury F., Charrier B., Bahabri F., Ganash A. (2014). MALDI-TOF Analysis of Aleppo Pine (*Pinus halepensis*) bark tannin. BioResources.

[46] Ucar M.B., Ucar G., Pizzi A., Gonultas O. (2013). Characterization of *Pinus brutia* bark tannin by MALDI-TOF MS and ^13^C NMR. Ind. Crops Prod..

[47] Saad H., Charrier-El Bouhtoury F., Pizzi A., Rode K., Charrier B., Ayed N. (2012). Characterization of pomegranate peels tannin extractives. Ind. Crops Prod..

[48] Hundt H.K.L., Roux D.G. (1978). Condensed tannins: Determination of the point of linkage in ‘terminal’(+)-catechin units and degradative bromination of 4-flavanylflavan-3,4-diols. J. Chem. Soc. Chem. Commun..

[49] Botha J.J., Ferreira D., Roux D.G. (1978). Condensed tannins. Circular dichroism method of assessing the absolute configuration at C-4 of 4-arylflavan-3-ols, and stereochemistry of their formation from flavan-3,4-diols. J. Chem. Soc. Chem. Commun..

[50] Basso M.C., Lacoste C., Pizzi A., Fredon E., Delmotte L. (2014). MALDI-TOF and ^13^C NMR analysis of flexible films and lacquers derived from tannin. Ind. Crops Prod..

[51] Delgado-Sánchez C., Amaral-Labat G., Grishechko L.I., Sánchez–Sánchez A., Fierro V., Pizzi A., Celzard A. (2017). Fire-resistant tannin–ethylene glycol gels working as rubber springs with tuneable elastic properties. J. Mater. Chem. A.

[52] Brinker C.J., Scherer G.W. (1990). Sol.—gel science. The physics and chemistry of Sol.—gel processing.

[53] Szczurek A., Amaral-Labat G., Fierro V., Pizzi A., Celzard A. (2011). The use of tannin to prepare carbon gels. Part II. Carbon cryogels. Carbon.

[54] Amaral-Labat G., Grishechko L., Szczurek A., Fierro V., Pizzi A., Kuznetsov B., Celzard A. (2012). Highly mesoporous organic aerogels derived from soy and tannin. Green Chem..

[55] Hench L.L., West J.K. (1990). The sol-gel process. Chem. Rev..

[56] Pekala R.W., Schaefer D.W. (1993). Structure of organic aerogels. 1. Morphology and scaling. Macromolecules.

[57] Job N., Pirard R., Marien J., Pirard J.-P. (2004). Porous carbon xerogels with texture tailored by pH control during sol–gel process. Carbon.

[58] Wang J., Glora M., Petricevic R., Saliger R., Proebstle H., Fricke J. (2001). Carbon cloth reinforced carbon aerogel films derived from resorcinol formaldehyde. J. Porous Mater..

[59] Bock V., Emmerling A., Saliger R., Fricke J. (1997). Structural investigation of resorcinol formaldehyde and carbon aerogels using SAXS and BET. J. Porous Mater..

[60] Amaral-Labat G., Szczurek A., Fierro V., Pizzi A., Celzard A. (2013). Systematic studies of tannin–formaldehyde aerogels: Preparation and properties. Sci. Technol. Adv. Mater..

[61] Amaral-Labat G., Grishechko L.I., Fierro V., Kuznetsov B.N., Pizzi A., Celzard A. (2013). Tannin-based xerogels with distinctive porous structures. Biomass Bioenergy.

[62] Job N., Panariello F., Marien J., Crine M., Pirard J.-P., Léonard A. (2006). Synthesis optimization of organic xerogels produced from convective air-drying of resorcinol–formaldehyde gels. J. Non-Cryst. Solids.

[63] Job N., Théry A., Pirard R., Marien J., Kocon L., Rouzaud J.-N., Béguin F., Pirard J.-P. (2005). Carbon aerogels, cryogels and xerogels: Influence of the drying method on the textural properties of porous carbon materials. Carbon.

[64] Castro C.D., Quintana G.C. (2015). Mixture design approach on the physical properties of lignin-resorcinol-formaldehyde xerogels. Int. J. Polym. Sci..

[65] Aegerter M.A., Leventis N., Koebel M.M. (2011). Aerogels Handbook. Advances in Sol-Gel Derived Materials and Technologies.

[66] García-González C.A., Alnaief M., Smirnova I. (2011). Polysaccharide-based aerogels—Promising biodegradable carriers for drug delivery systems. Carbohydr. Polym..

[67] Robb S.A., Lee B.H., McLemore R., Vernon B.L. (2007). Simultaneously physically and chemically gelling polymer system utilizing a poly(NIPAAm-co-cysteamine)-based copolymer. Biomacromolecules.

[68] Pizzi A., Scharfetter H.O. (1978). The chemistry and development of tannin-based adhesives for exterior plywood. J. Appl. Polym. Sci..

[69] Ping L., Brosse N., Chrusciel L., Navarrete P., Pizzi A. (2011). Extraction of condensed tannins from grape pomace for use as wood adhesives. Ind. Crops Prod..

[70] Pizzi A. (1983). Wood Adhesive Chemistry and Technology.

[71] Fraser D.A., Hall R.W., Raum A.L.J. (2007). Preparation of ‘high-ortho’ novolak resins I. Metal ion catalysis and orientation effect. J. Appl. Chem..

[72] Fraser D.A., Hall R.W., Jenkins P.A., Raum A.L.J. (2007). Preparation of ‘high-ortho’ novolak resins. II. The course of the reaction. J. Appl. Chem..

[73] Pizzi A. (1979). Phenolic resins by reactions of coordinated metal ligands. J. Polym. Sci. Polym. Lett. Ed..

[74] Pizzi A. (1979). Phenolic and tannin-based adhesive resins by reactions of coordinated metal ligands. I. Phenolic chelates. J. Appl. Polym. Sci..

[75] Pizzi A. (1979). Phenolic and tannin-based adhesive resins by reactions of coordinated metal ligands. II. Tannin adhesive preparation, characteristics, and application. J. Appl. Polym. Sci..

[76] Hillis W.E., Urbach G. (2007). The reaction of (+)-catechin with formaldehyde. J. Appl. Chem..

[77] Szczurek A., Amaral-Labat G., Fierro V., Pizzi A., Masson E., Celzard A. (2011). The use of tannin to prepare carbon gels. Part I: Carbon aerogels. Carbon.

[78] Grishechko L.I., Amaral-Labat G., Szczurek A., Fierro V., Kuznetsov B.N., Pizzi A., Celzard A. (2013). New tannin–lignin aerogels. Ind. Crops Prod..

[79] Amaral-Labat G.A., Pizzi A., Gonçalves A.R., Celzard A., Rigolet S., Rocha G.J.M. (2008). Environment-friendly soy flour-based resins without formaldehyde. J. Appl. Polym. Sci..

[80] Lacoste C., Basso M.C., Pizzi A., Celzard A., Laborie M.-P. (2015). Natural albumin/tannin cellular foams. Ind. Crops Prod..

[81] Yoshizawa N., Hatori H., Soneda Y., Hanzawa Y., Kaneko K., Dresselhaus M.S. (2003). Structure and electrochemical properties of carbon aerogels polymerized in the presence of Cu^2+^. J. Non-Cryst. Solids.

[82] Amaral-Labat G., Szczurek A., Fierro V., Pizzi A., Masson E., Celzard A. (2012). “Blue glue”: A new precursor of carbon aerogels. Microporous Mesoporous Mater..

[83] Nakano Y., Takeshita K., Tsutsumi T. (2001). Adsorption mechanism of hexavalent chromium by redox within condensed-tannin gel. Water Res..

[84] Zhan X.-M., Zhao X. (2003). Mechanism of lead adsorption from aqueous solutions using an adsorbent synthesized from natural condensed tannin. Water Res..

[85] Ogata T., Nakano Y. (2005). Mechanisms of gold recovery from aqueous solutions using a novel tannin gel adsorbent synthesized from natural condensed tannin. Water Res..

[86] Kim Y.-H., Ogata T., Nakano Y. (2007). Kinetic analysis of palladium(II) adsorption process on condensed-tannin gel based on redox reaction models. Water Res..

[87] Şengil İ.A., Özacar M. (2008). Biosorption of Cu(II) from aqueous solutions by mimosa tannin gel. J. Hazard. Mater..

[88] Sánchez-Martín J., Beltrán-Heredia J., Gibello-Pérez P. (2011). Adsorbent biopolymers from tannin extracts for water treatment. Chem. Eng. J..

[89] Al-Muhtaseb S.A., Ritter J.A. (2003). Preparation and properties of resorcinol-formaldehyde organic and carbon gels. Adv. Mater..

[90] García-González C.A., Camino-Rey M.C., Alnaief M., Zetzl C., Smirnova I. (2012). Supercritical drying of aerogels using CO_2_: Effect of extraction time on the end material textural properties. J. Supercrit. Fluids.

[91] Fricke J., Tillotson T. (1997). Aerogels: Production, characterization, and applications. Thin Solid Films.

[92] Amaral-Labat G. (2013). Gels Poreux Biosourcés: Production, Caractérisation et Applications. Doctoral Dissertation.

[93] Scherer G.W., Smith D.M. (1995). Cavitation during drying of a gel. J. Non-Cryst. Solids.

[94] Daraoui N., Dufour P., Hammouri H., Hottot A. (2010). Model predictive control during the primary drying stage of lyophilisation. Control. Eng. Pract..

[95] Tamon H., Ishizaka H., Yamamoto T., Suzuki T. (1999). Preparation of mesoporous carbon by freeze drying. Carbon.

[96] Baetens R., Jelle B.P., Gustavsen A. (2011). Aerogel insulation for building applications: A state-of-the-art review. Energy Build..

[97] Rey-Raap N., Szczurek A., Fierro V., Celzard A., Menéndez J.A., Arenillas A. (2016). Advances in tailoring the porosity of tannin-based carbon xerogels. Ind. Crops Prod..

[98] Amaral-Labat G., Szczurek A., Fierro V., Celzard A. (2015). Unique bimodal carbon xerogels from soft templating of tannin. Mater. Chem. Phys..

[99] Braghiroli F.L., Fierro V., Parmentier J., Pasc A., Celzard A. (2016). Easy and eco-friendly synthesis of ordered mesoporous carbons by self-assembly of tannin with a block copolymer. Green Chem..

[100] Libra J.A., Ro K.S., Kammann C., Funke A., Berge N.D., Neubauer Y., Titirici M.-M., Fühner C., Bens O., Kern J. (2011). Hydrothermal carbonization of biomass residuals: A comparative review of the chemistry, processes and applications of wet and dry pyrolysis. Biofuels.

[101] Brun N., García-González C.A., Smirnova I., Titirici M.M. (2013). Hydrothermal synthesis of highly porous carbon monoliths from carbohydrates and phloroglucinol. RSC Adv..

[102] Fellinger T.-P., White R.J., Titirici M.-M., Antonietti M. (2012). Borax-mediated formation of carbon aerogels from glucose. Adv. Funct. Mater..

[103] Wohlgemuth S.-A., White R.J., Willinger M.-G., Titirici M.-M., Antonietti M. (2012). A one-pot hydrothermal synthesis of sulfur and nitrogen doped carbon aerogels with enhanced electrocatalytic activity in the oxygen reduction reaction. Green Chem..

[104] White R.J., Yoshizawa N., Antonietti M., Titirici M.-M. (2011). A sustainable synthesis of nitrogen-doped carbon aerogels. Green Chem..

[105] Braghiroli F.L., Fierro V., Izquierdo M.T., Parmentier J., Pizzi A., Celzard A. (2012). Nitrogen-doped carbon materials produced from hydrothermally treated tannin. Carbon.

[106] Braghiroli F.L., Fierro V., Parmentier J., Vidal L., Gadonneix P., Celzard A. (2015). Hydrothermal carbons produced from tannin by modification of the reaction medium: Addition of H^+^ and Ag^+^. Ind. Crops Prod..

[107] Navarrete P., Pizzi A., Bertaud F., Rigolet S. (2011). Condensed tannin reactivity inhibition by internal rearrangements: Detection by CP-MAS ^13^C NMR. Maderas Cienc. Tecnol..

[108] Young D.A., Cronjé A., Botes A.L., Ferreira D., Roux D.G. (1985). Synthesis of condensed tannins. Part 14. Biflavanoid profisetinidins as synthons. The Acid-induced ‘phlobaphene’ reaction. J. Chem. Soc. Perkin Trans..

[109] Braghiroli F.L., Fierro V., Izquierdo M.T., Parmentier J., Pizzi A., Delmotte L., Fioux P., Celzard A. (2015). High surface—highly N-doped carbons from hydrothermally treated tannin. Ind. Crops Prod..

[110] Braghiroli F., Fierro V., Pizzi A., Rode K., Radke W., Delmotte L., Parmentier J., Celzard A. (2013). Reaction of condensed tannins with ammonia. Ind. Crops Prod..

[111] Braghiroli F.L., Fierro V., Szczurek A., Stein N., Parmentier J., Celzard A. (2015). Hydrothermally treated aminated tannin as precursor of N-doped carbon gels for supercapacitors. Carbon.

[112] Deng X., Liu X., Duan T., Zhu W., Yi Z., Yao W. (2016). Fabricating a graphene oxide—bayberry tannin sponge for effective radionuclide removal. Mater. Res. Express.

[113] Braghiroli F., Fierro V., Szczurek A., Gadonneix P., Ghanbaja J., Parmentier J., Medjahdi G., Celzard A. (2017). Hydrothermal treatment of tannin: A route to porous metal oxides and metal/carbon hybrid materials. Inorganics.

[114] Braghiroli F.L. (2014). Polyphénols Végétaux Traités par Voie Humide: Synthèse de Carbones Biosourcés Hautement Poreux et Applications. Doctoral Dissertation.

[115] Pandey A., Bhaskar T., Stöcker M., Sukumaran R. (2015). Recent Advances in Thermochemical Conversion of Biomass.

[116] Marsh H., Rodríguez-Reinoso F. (2006). Activated Carbon.

[117] Marsh H., Rodríguez-Reinoso F. (2006). Chapter 5—Activation Processes (Thermal or Physical). Activated Carbon.

[118] Szczurek A., Amaral-Labat G., Fierro V., Pizzi A., Celzard A. (2014). Chemical activation of tannin-based hydrogels by soaking in KOH and NaOH solutions. Microporous Mesoporous Mater..

[119] Marsh H., Rodríguez-Reinoso F. (2006). Chapter 6—Activation Processes (Chemical). Activated Carbon.

[120] Reimerink W.M.T.M. (1999). The use of activated carbon as catalyst and catalyst carrier in industrial applications. Studies in Surface Science and Catalysis.

[121] Chen J.Y. (2017). Activated Carbon Fiber and Textiles.

[122] Wu D., Fu R., Sun Z., Yu Z. (2005). Low-density organic and carbon aerogels from the sol–gel polymerization of phenol with formaldehyde. J. Non-Cryst. Solids.

[123] Szczurek A., Amaral-Labat G., Fierro V., Pizzi A., Masson E., Celzard A. (2011). Porosity of resorcinol-formaldehyde organic and carbon aerogels exchanged and dried with supercritical organic solvents. Mater. Chem. Phys..

[124] Rey-Raap N., Szczurek A., Fierro V., Menéndez J.A., Arenillas A., Celzard A. (2015). Towards a feasible and scalable production of bio-xerogels. J. Colloid Interface Sci..

[125] Grishechko L.I., Amaral-Labat G., Fierro V., Szczurek A., Kuznetsov B.N., Celzard A. (2016). Biosourced, highly porous, carbon xerogel microspheres. RSC Adv..

[126] Amaral-Labat G., Szczurek A., Fierro V., Stein N., Boulanger C., Pizzi A., Celzard A. (2012). Pore structure and electrochemical performances of tannin-based carbon cryogels. Biomass Bioenergy.

[127] Szczurek A., Fierro V., Medjahdi G., Celzard A. (2019). Carbon aerogels prepared by autocondensation of flavonoid tannin. Carbon Resour. Convers..

[128] Sanchez-Sanchez A., Izquierdo M.T., Mathieu S., González-Álvarez J., Celzard A., Fierro V. (2017). Outstanding electrochemical performance of highly N- and O-doped carbons derived from pine tannin. Green Chem..

[129] Fernández-Barbero A., Suárez I.J., Sierra-Martín B., Fernández-Nieves A., de las Nieves F.J., Marquez M., Rubio-Retama J., López-Cabarcos E. (2009). Gels and microgels for nanotechnological applications. Adv. Colloid Interface Sci..

[130] Braghiroli F.L., Bouafif H., Neculita C.M., Koubaa A. (2018). Activated biochar as an effective sorbent for organic and inorganic contaminants in water. Water. Air. Soil Pollut..

[131] Bi Z., Kong Q., Cao Y., Sun G., Su F., Wei X., Li X., Ahmad A., Xie L., Chen C.-M. (2019). Biomass-derived porous carbon materials with different dimensions for supercapacitor electrodes: A review. J. Mater. Chem. A.

[132] Frackowiak E., Béguin F. (2001). Carbon materials for the electrochemical storage of energy in capacitors. Carbon.

[133] Hsieh C.-T., Teng H. (2002). Influence of oxygen treatment on electric double-layer capacitance of activated carbon fabrics. Carbon.

[134] Wei L., Sevilla M., Fuertes A.B., Mokaya R., Yushin G. (2011). Hydrothermal carbonization of abundant renewable natural organic chemicals for high-performance supercapacitor electrodes. Adv. Energy Mater..

[135] Zhao L., Fan L.-Z., Zhou M.-Q., Guan H., Qiao S., Antonietti M., Titirici M.-M. (2010). Nitrogen-containing hydrothermal carbons with superior performance in supercapacitors. Adv. Mater..

[136] Si W., Zhou J., Zhang S., Li S., Xing W., Zhuo S. (2013). Tunable N-doped or dual N, *S*-doped activated hydrothermal carbons derived from human hair and glucose for supercapacitor applications. Electrochim. Acta.

[137] Zapata-Benabithe Z., Diossa G., Castro C.D., Quintana G. (2016). Activated carbon bio-xerogels as electrodes for supercapacitors applications. Procedia Eng..

[138] Alvares Rodrigues L., Koibuchi Sakane K., Alves Nunes Simonetti E., Patrocínio Thim G. (2015). Cr total removal in aqueous solution by PHENOTAN AP based tannin gel (TFC). J. Environ. Chem. Eng..

[139] Kunnambath P.M., Thirumalaisamy S. (2015). Characterization and utilization of tannin extract for the selective adsorption of Ni(II) ions from water. J. Chem..

[140] Nakajima A. (2002). Electron spin resonance study on the vanadium adsorption by persimmon tannin gel. Talanta.

[141] Nakajima A., Ohe K., Baba Y., Kijima T. (2003). Mechanism of gold adsorption by persimmon tannin gel. Anal. Sci. Int. J. Jpn. Soc. Anal. Chem..

[142] Kim Y.-H., Alam M.N., Marutani Y., Ogata T., Morisada S., Nakano Y. (2009). Improvement of Pd(II) adsorption performance of condensed-tannin gel by amine modification. Chem. Lett..

[143] Morisada S., Kim Y.-H., Ogata T., Marutani Y., Nakano Y. (2011). Improved adsorption behaviors of amine-modified tannin gel for palladium and platinum ions in acidic chloride solutions. Ind. Eng. Chem. Res..

[144] Ogata T., Kim Y.H., Nakano Y. (2007). Selective recovery process for gold utilizing a functional gel derived from natural condensed tannin. J. Chem. Eng. Jpn..

[145] Gurung M., Adhikari B.B., Kawakita H., Ohto K., Inoue K., Alam S. (2012). Selective recovery of precious metals from acidic leach liquor of circuit boards of spent mobile phones using chemically modified Persimmon tannin gel. Ind. Eng. Chem. Res..

[146] Morisada S., Rin T., Ogata T., Kim Y.-H., Nakano Y. (2011). Adsorption removal of boron in aqueous solutions by amine-modified tannin gel. Water Res..

[147] Ogata T., Morisada S., Oinuma Y., Seida Y., Nakano Y. (2011). Preparation of adsorbent for phosphate recovery from aqueous solutions based on condensed tannin gel. J. Hazard. Mater..

[148] Sánchez-Martín J., González-Velasco M., Beltrán-Heredia J., Gragera-Carvajal J., Salguero-Fernández J. (2010). Novel tannin-based adsorbent in removing cationic dye (Methylene Blue) from aqueous solution. Kinetics and equilibrium studies. J. Hazard. Mater..

[149] Rahman M., Akter N., Karim M.R., Ahmad N., Rahman M.M., Siddiquey I.A., Bahadur N.M., Hasnat M.A. (2014). Optimization, kinetic and thermodynamic studies for removal of Brilliant Red (X-3B) using Tannin gel. J. Environ. Chem. Eng..

[150] Fayemiwo O.M., Daramola M.O., Moothi K. (2018). Tannin-based adsorbents from green tea for removal of monoaromatic hydrocarbons in water: Preliminary investigations. Chem. Eng. Commun..

